# Effects of Naturally Occurring Mutations in Bovine Leukemia Virus 5′-LTR and Tax Gene on Viral Transcriptional Activity

**DOI:** 10.3390/pathogens9100836

**Published:** 2020-10-13

**Authors:** Aneta Pluta, Luc Willems, Renée N. Douville, Jacek Kuźmak

**Affiliations:** 1 Department of Biochemistry, National Veterinary Research Institute, 24-100 Puławy, Poland; jkuzmak@piwet.pulawy.pl; 2 Molecular and Cellular Epigenetics (Interdisciplinary Cluster for Applied Genoproteomics, GIGA) and Molecular Biology (TERRA), University of Liège (ULiège), 4000 Liege, Belgium; luc.willems@uliege.be; 3 Department of Biology, The University of Winnipeg, Winnipeg, MB R3B 2E9, Canada; r.douville@uwinnipeg.ca; 4 Department of Immunology, University of Manitoba, Winnipeg, MB R3E 0T5, Canada

**Keywords:** retrovirus, bovine leukemia virus (BLV), long terminal repeat (LTR), Tax protein, transactivation, transcription factor, proviral load, sequence variants, primary isolates

## Abstract

Bovine leukemia virus (BLV) is a deltaretrovirus infecting bovine B cells and causing enzootic bovine leucosis (EBL). The long terminal repeat (LTR) plays an indispensable role in viral gene expression. The BLV Tax protein acts as the main transactivator of LTR-driven transcription of BLV viral genes. The aim of this study was to analyze mutations in the BLV LTR region and *tax* gene to determine their association with transcriptional activity. LTRs were obtained from one hundred and six BLV isolates and analyzed for their genetic variability. Fifteen variants were selected and characterized based on mutations in LTR regulatory elements, and further used for in vitro transcription assays. Reporter vectors containing the luciferase gene under the control of each variant BLV promoter sequence, in addition to variant Tax expression vectors, were constructed. Both types of plasmids were used for cotransfection of HeLa cells and the level of luciferase activity was measured as a proxy of transcriptional activity. Marked differences in LTR promoter activity and Tax transactivation activity were observed amongst BLV variants. These results demonstrate that mutations in both the BLV LTR and *tax* gene can affect the promoter activity, which may have important consequences on proviral load, viral fitness, and transmissibility in BLV-infected cattle.

## 1. Introduction

Bovine leukemia virus (BLV), which belongs to the *Deltaretrovirus* genus of *Retroviridae* family, is the etiologic agent of enzootic bovine leucosis (EBL). EBL is a lymphoproliferative disease characterized by B-cell lymphoma, and occurs in cattle throughout the world [1,2,3,4,5]. In the majority of cases, infection is asymptomatic; but, approximately one-third of BLV-infected cattle develop persistent lymphocytosis and less than 5% progress to B-cell lymphoma [6]. There is no treatment for viral infection. Eradication programs have been developed but success has been variable, primarily because of the expense and high prevalence of infection among cattle in the particular countries of the Americas, Eurasia, and Asia.

The BLV genome is comprised of two identical copies of single-stranded RNA. This viral RNA is reverse transcribed to form a double-stranded DNA called provirus, flanked by long terminal repeats (LTRs) [7,8]. The provirus contains the genes encoding capsid (*gag*) and envelope proteins (*env*), as well as enzymes (*pol*), accessory proteins, and microRNAs [9,10,11]. The LTR contains the promoter required for virus gene expression [12]. The LTR is composed of the U3, R, and U5 regions, wherein the U3 encompasses critical transcription factor (TF) binding sites for transcription initiation. Transcription of BLV genes is initiated at the U3/R junction in the 5′-LTR [13]. The U3 region includes the three TREs (Tax Responsive Elements) which carry three imperfectly conserved 21-bp enhancer elements—named viral cAMP-response elements (CREs)—that bind cAMP-responsive element-binding protein/activating transcription factor (CREB/ATF) family members [14,15]. The virus-encoded Tax protein is a transactivator which increases the DNA binding activity of the CREB/ATF proteins by interacting with their bZip domains and positively regulating activation of BLV transcription [16,17]. Besides the CRE elements, each TRE sequence includes an E box as a target sequence for the AP4 transcription factor. Additionally, the U3 region contains several other response elements, such as a nuclear factor-κB (κB) binding site, a glucocorticoid response element (GRE), and PU.1/Spi-B binding sites for ETS (erythroblast transformation specific) transcription factor family proteins [18,19,20]. Additional elements of transcription control have been identified within R and U5 sequences of 5′ LTR. At least three different elements—an upstream stimulatory factor (USF) binding site, downstream activator sequence (DAS), and an interferon regulatory factor (IRF) binding site—have been identified [21,22]. 

The genome of BLV is remarkably stable [23]. To date, all strains which have been sequenced in their entirety have exhibited 94.8% to 99.5% similarity in their nucleotide sequence. Direct sequencing of PCR-amplified fragments of the BLV genome from BLV-infected cattle have validated this observation [24,25]. Although most of the BLV genome appears conserved, considerable variations are observed in select regions. So far, current opinion is that sequence variations are a reflection of the geographical origin of the isolate, more so than dictated by mutational pressure from BLV pathology [5,26,27,28,29,30,31,32]. However, we do not know whether the small number of point mutations that distinguish different isolates have functional significance. Many of these mutations have been found in the LTR region containing the viral promoter and enhancers [24,33]. In case of murine leukemia virus (MLV), a spontaneous mutation at position −166 in the U3 region which generates a novel Sp1 binding site was shown to increase transcription and enable viral replication in embryonic cells [34]. Similarly, in human T-cell lymphotrophic virus (HTLV-1), naturally occurring mutations in the LTR R region affects ATF2 binding among HTLV-1-associated myelopathy/tropical spastic paraparesis (HAM/TSP) patients [35]. Only few studies evaluated BLV LTR sequences for the presence of single nucleotide polymorphisms (SNPs) and their impact on virus replication. In our previous paper, SNPs were detected mostly in the U3 region of LTR, followed by U5 and then the R region [36]. SNPs in the U3 region of the LTR occurred within and outside enhancer and promoter sequences. Thus, it is possible that mutations found in the LTR are responsible for the variable aftermaths of virus infection. Indeed, spontaneous substitution of T175C within TATA box in U3 region of the 5’-LTR was associated with virus productivity in vitro, which closely correlates with BLV transmissibility [33]. 

Expression of BLV is regulated by the viral transcriptional activator Tax. Apart from its interaction with CREB/ATF family members, BLV Tax can also interact with histone acetyltransferases to facilitate BLV transcription through targeted acetylation of nucleosomal histones. Known functional domains of the Tax protein include a putative zinc finger motif, a leucine-rich activation domain, a multifunctional domain, and two phosphorylation sites. Given the important role of Tax in viral replication, surprisingly little is known of its sequence variation. Polat et al. found specific mutations P(100)S and F(108)L in the Tax protein that were observed only in samples from South American BLV strains [31]. Zyrianova et al. showed that Tax variants with a E(51)G mutation in the putative zinc finger domain were associated with an increase of white blood cell count in BLV-infected cattle in the persistent lymphocytosis stage [37]. In addition, several *tax* gene nucleotide mutations were found to compromise viral protein function and contribute to reduced transactivation activity [38,39,40]. A naturally occurring E(303)K change in Tax impaired its function, resulting in reduced transcriptional activity of the LTR promoter in vitro and induced the silent BLV phenotype in a BLV-induced B-cell tumor [41,42]. The defect could be compensated by reverse genetics [43]. It was also shown that amino acid changes between residues 240 and 265 of Tax resulted in a significant increase of its transactivation activity by engaging a CRE motif in the BLV LTR sequence [44]. A S(240)P substitution exhibited an enhanced ability to reduce viral LTR-driven transcription compared to wild type protein, while D(247)G enhanced viral expression and propagation in vitro [44]. Therefore, it is suspected that *tax* sequence variation may have a substantial impact on the transcriptional activity, inactivation, or virus silencing. 

In this paper, we sequenced both LTR and Tax gene sequences from 106 BLV isolates and analyzed their impact on BLV promoter activity. 

## 2. Results

### 2.1. Sequence Variations in BLV LTR Regions 

To identify the extent of genetic variation within the 106 BLV isolates, a multiple alignment of the LTR sequences, with respect to reference BLV strain 344, was performed using ClustalW algorithm (Figure S1). The analysis revealed a 98.2% averaged pairwise identity. Point mutations were found among all sequences. Similar sequence variation was observed in isolates derived from the same cattle herd, as well as different herds. The most frequent changes in the U3 region included the following substitutions: A(−137)G and A(−135)G in κB-like site (5/106); C(−65)T in the GRE (43/106); G(−43)T in TATA box (5/106); substitution of T(−41)A in the TATA Box (12/106); and A(−37)T and C(−36)T in the TATA Box binding protein site, observed in 92 and 63 sequences, respectively. Additionally, the LTR sequence analysis revealed the presence of one deletion located in T(−11)del of CAP site in 8 sequences. The most frequent changes observed in regulatory elements of the R and U5 regions were characterized by substitution of A(+150)G in box A of the DAS region (12/106), substitution TC(+188/9)CT in box C of DAS (22/106) and substitution T(+190)C in box C (14/106). Additionally, a double insertion CT(+191/2)del of box C (10/106) was noted. In the U5 region a T(+256)C transition in IRF occurred in 3 sequences (Figure S1).

Based on the multiple alignment of these 106 sequences and two reference sequences (BLV strain 344 and BLV-FLK), a phylogenetic tree was constructed by use of a Bayesian method. The topology, with high posterior probabilities, indicated that all sequences were clearly classified into three distinct groups (Figure S2) corresponding to the G4, G7, and G8 genotypes [36]. The seventy-four sequences were clustered within G4 genotype with a moderate posterior probability of 0.57. Further, they were separated into distinct subgroups, designated as G4-Ia-e, G4-II, and G4-IIIa-c. The twenty-one sequences were grouped into genotype G8, creating subgroup G8-I with high posterior value = 1.00. The remaining eleven sequences were found to create a G7-I subgroup of the genotype G7 with high posterior value = 1.00. 

### 2.2. BLV LTR Variants Are Determined by Specific Mutation Patterns

**Figure 1:**
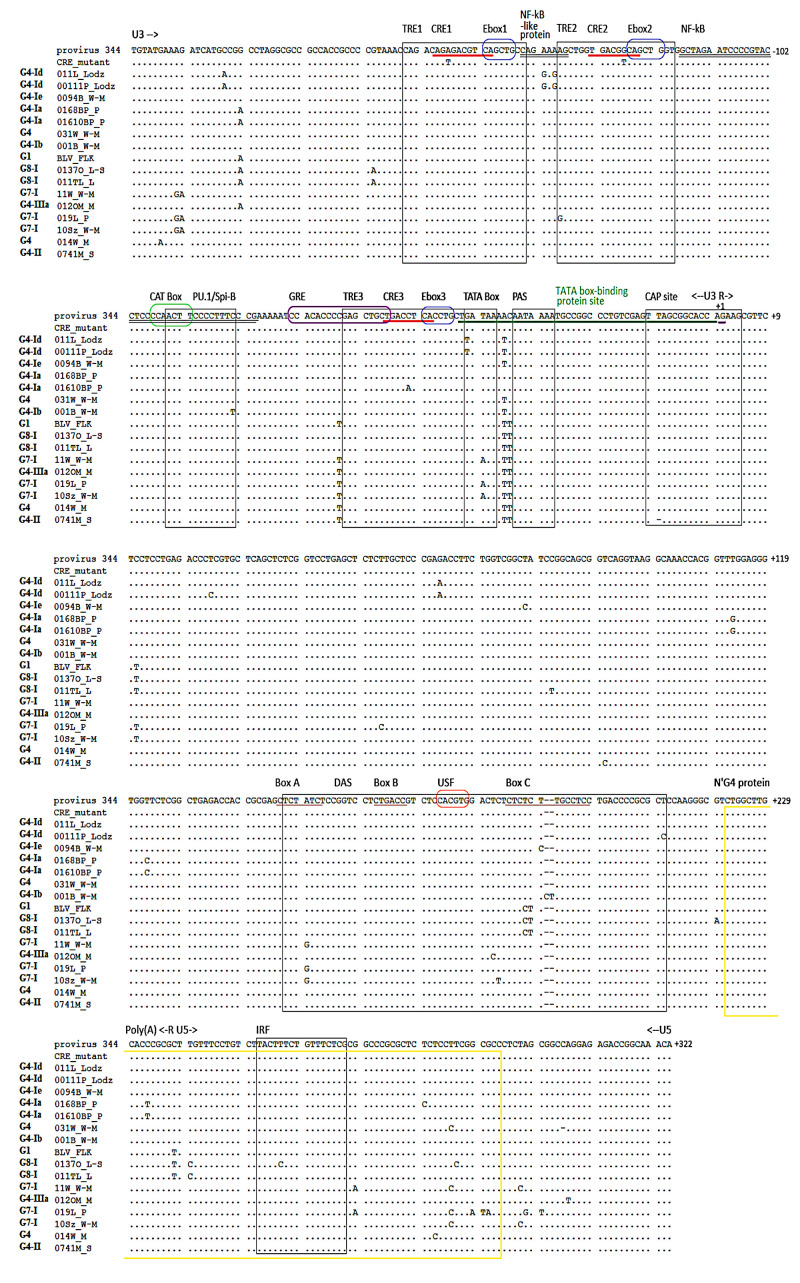
Alignment of the nucleotide sequences of fifteen bovine leukemia virus (BLV) long terminal repeat (LTR) variants. Differences from the reference strain 344 sequence (JC613347.1) are indicated, as is the distribution of corresponding regulatory elements along the LTR. TxRE1, 2, and 3, TATA Box, PU.1/Spi-B, polyadenylation site (PAS) and downstream activator sequence (DAS) (Box A–C), catabolite activator protein (CAP) site, and interferon regulatory factor (IRF) are functionally important regions and are indicated with black boxes. Amino-terminal fragment of G4 protein is indicated with a yellow box. cAMP-response element (CRE) 1, 2 and 3 motifs are underlined with a red line; Ebox1, 2 and 3, CAT Box, glucocorticoid response element (GRE), and upstream stimulatory factor (USF) motif are marked with blue, green, violet, and orange oval rectangles, respectively; κB, NF-κB-like protein, and TATA box-binding protein site are underlined by a double black line and green line, respectively. Assignment of particular variants to genotypes and subtypes is indicated on the left side of the alignment. The boundaries of the U3, R, and U5 sequences are indicated by arrows.

Fifteen representative LTR sequence variants were selected for further study based on the following criteria: (1) affiliation of particular variants to major subtype and genotype based on phylogenetic study; (2) harboring mutations most commonly observed in regulatory elements of LTR; and, (3) frequency of particular mutations within collected isolates. Figure 1 shows a nucleotide sequence alignment with illustration of particular changes based on the above selection parameters. Select SNPs in promoter and regulatory sites characteristic for each given LTR variant are shown in Table 1. These 15 LTR variants are representative of 79 of the 106 mapped LTR sequences (i.e., up to 74.5% of all sequences). Importantly, sequences with mutations in nonregulatory sites, for which in-silico analysis indicated new putative transcription factor binding sites, were not assigned among these 15 representative variants.

### 2.3. Increased Basal Transcriptional Activity of BLV LTRs Harboring SNPs in Regulatory Regions

**Figure 2:**
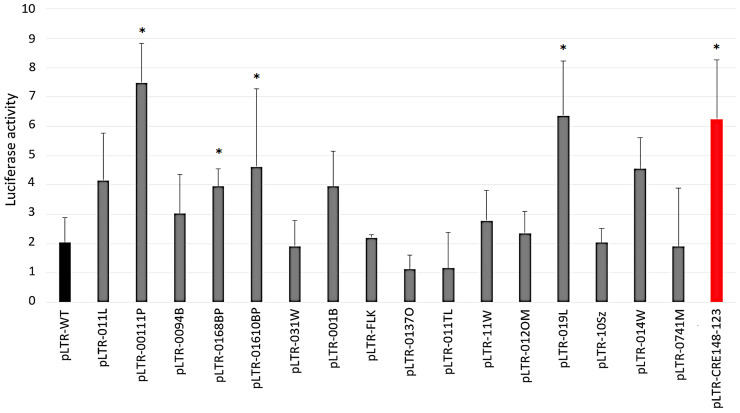
Basal transcriptional activity of BLV LTR variants and a representative strain BLV LTR. Fifteen luciferase-based plasmids (pLTR-011L, pLTR-00111P, pLTR-0094B, pLTR-0168BP, pLTR-01610BP, pLTR-031W, pLTR-001B, pLTR-0137O, pLTR-011TL, pLTR-11W, pLTR-012OM, pLTR-019L, pLTR-10Sz, pLTR-014W, and pLTR-0741M) and pLTR-FLK (pLTR-FLK plasmid includes LTR sequence of strain BLV913; EF600696.1 [45]) are marked in gray. pLTR-WT and pLTR-CRE148-123 were transfected into HeLa cells in absence of Tax. Vectors pLTR-WT (marked in black) and pLTR-CRE148-123 [15] (marked in red) were used as reference and positive control, respectively. All these reporter data from cell culture were averages from three independent experiments. * Statistically significant by Mann–Whitney U Test (*p* < 0.05). Error bars represent standard deviations.

To evaluate the effect of basal transcriptional activity in the selected variants, 15 full-length LTR sequences were subcloned into the pGL4.11 vector, a luciferase-based reporter. The resulting plasmids together with a Renilla reference vector were cotransfected into HeLa cells. Plasmids pLTR-0094B, pLTR-031W, pLTR-FLK, pLTR-0137O, pLTR-011TL, pLTR-11W, pLTR-012OM, pLTR-10Sz, and pLTR-0741M yielded luciferase activities comparable to reference (pLTR-WT; Figure 2).

Transfections with plasmids pLTR-011L, pLTR-0168BP, pLTR-001B, and pLTR-014W revealed slightly increased luciferase activity (2-fold enhancement). The difference in luciferase activity level between the pLTR-WT and different pLTRs was analyzed by Mann–Whitney U test and *p*-values are shown in Table S1. HeLa cells transfected with plasmids pLTR-00111P, pLTR-01610BP, and pLTR-019L showed notably increased luciferase activity (2.3–3.7-fold) (Figure 2, Table S1). These three LTR variants harbored notable mutations, A(−137)G, A(−135)G, and A(−134)G in κB-like site; G(−43)T in TATA box; C(-53)A in CRE3; and T(+113)G in putative Myc-associated zinc finger protein (MAZ).

### 2.4. Transcriptional Activities of BLV LTR Variants in the Presence of Tax344 Expression Plasmid

**Figure 3:**
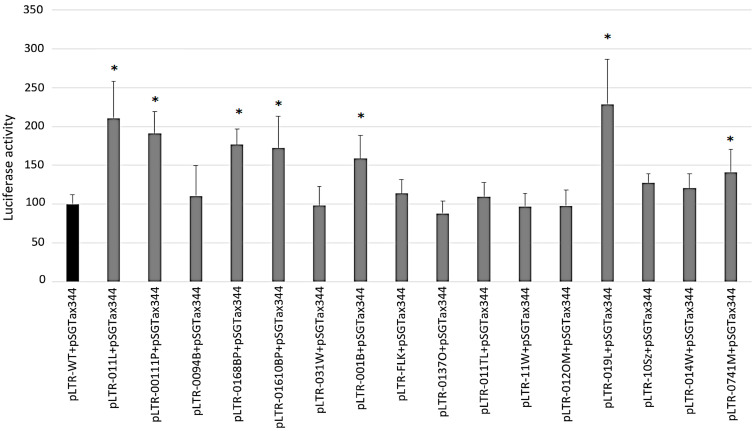
Promoter activity from the interaction of BLV LTR variants with reference strain BLV Tax. HeLa cells were cotransfected with luciferase-based plasmids (pLTR-011L, pLTR-00111P, pLTR-0094B, pLTR-0168BP, pLTR-01610BP, pLTR-031W, pLTR-001B, pLTR-0137O, pLTR-011TL, pLTR-11W, pLTR-012OM, pLTR-019L, pLTR-10Sz, pLTR-014W, pLTR-0741M, pLTR-FLK, and pLTR-WT reference) and pSGTax344 344 reference strain Tax. All these reporter data from cell culture were averages from three independent experiments. * Statistically significant by Mann–Whitney U Test (*p* < 0.05). Error bars represent standard deviations.

Subsequently, promoter activity of the LTR variants were examined in the presence of the wild type Tax protein, by cotransfection with the pSGTax344 expression plasmid in HeLa cells. Although the variation in Tax-driven promoter activity (CV = 32.22974%) was lower than that of the LTR constructs transfected without Tax (CV = 52.03168%), this difference was not statistically significant based on the Wilcoxon signed-ranks test (*p* >0.05). In presence of Tax, the pLTR-0094B, pLTR-031W, pLTR-FLK, pLTR-0137O, pLTR-011TL, pLTR-11W, pLTR-012OM, pLTR-10Sz, and pLTR-014W plasmids generated luciferase activity at a level comparable to reference plasmids (pLTR-WT and pSGTax344) (Figure 3; Table S1). The pLTR-0741M plasmid slightly but reproducibly demonstrated increased LTR-directed luciferase activity (1.5-fold). However, the pLTR-011L, pLTR-00111P, pLTR-0168BP, pLTR-01610BP, pLTR-001B, and pLTR-019L plasmids significantly increased luciferase activity (1.6–2.4-fold). These six LTR variants harbored notable mutations—A(−137)G, A(-135)G, and A(−134)G in κB-like site; G(−43)T in TATA box; C(−53)A in CRE3; C(−83)T in PU.1/Spi-B; CT(+191/2)ins in box C of DAS; and T(+113)G in putative MAZ—that were not observed in the other BLV LTR variants.

### 2.5. Variations in BLV Tax Amino Acid Sequences 

Since the Tax protein acts through the 5′-LTR promoter to activate transcription initiation of BLV, the genetic diversity of the Tax amino acid sequence was evaluated. Forty-three genomic DNA samples were used for PCR amplification and all amplicons were successfully sequenced. The forty-three *tax* gene sequences, BLV-FLK (EF600696.1), and provirus 344 sequences (JC613347.1) were translated and the amino acid alignment is shown in Figure S3. Missense mutations from the provirus 344 amino acid sequence were found in 42 sequences. Of the 310 amino acids of the full-length Tax sequence, 30 sites with 34 different changes were identified. Out of the 34 changes, 19 were located within functional domains and epitopes [46,47,48,49,50]. A E(42)K change occurred in the Zn finger domain, S(124)F/P, I(130)L, S(140)N, L(141)V, and V(124)A changes occurred in a T-cell epitope site [47,48]. In the CD8+ CTL epitope, T(152)I, L(161)S, L(173)P, R(183)K, and I(186)T changes occurred, which encompass the leucine-rich activation domain and transcriptional activation domain within this epitope [49]. Tax C(257)G, C(257)Y, C(257)F, D(258)N, and S(265)G changes were located in multifunctional domain, while S(265)G, H(274)L, and L(278)I changes occurred in a B-cell epitope [48]. In comparison to the sequences of the polish isolates, the BLV-FLK Tax sequence was characterized by a distinct pattern of mutations along the whole protein (Figure S3). Notably, phosphorylation sites (S106 and S293) for all Tax variants were conserved.

**Figure 4:**
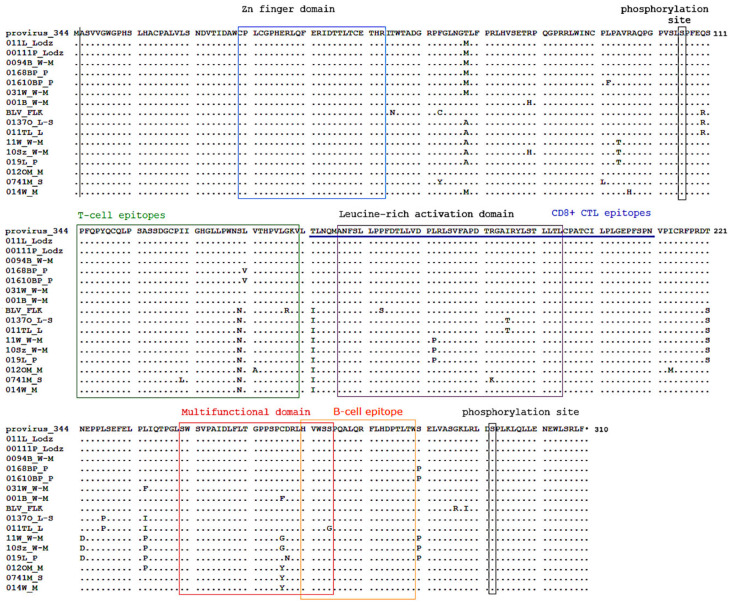
Alignment of the translated Tax protein amino acid sequences from fifteen select BLV variants. The points at which the translated amino acid sequence of the analyzed proviral Tax gene differ are indicated below the reference 344 strain sequence. Functionally important regions are identified with boxes: black denotes Zn finger domain and phosphorylation sites; green boxes denote T-cell epitopes and B-cell epitope; violet denotes Leucine-rich activation domain; red denotes the multifunctional domain. CD8+ CTL epitopes are underlined by a blue line.

A phylogenetic analysis based on the Tax amino acid sequences of the 43 polish BLV isolates with BLV-FLK and reference 344 isolate resulted in the classification of all sequences into four genotypes (G4, G7, G8, and G1; Figure S4). The fifteen corresponding *tax* gene sequences matched to the 15 LTR variants described above were selected for further study. The amino acid alignment of the selected BLV Tax amino acid sequences is shown in Figure 4. 

### 2.6. Increased Promoter Activity after Transactivation with BLV Tax Variants 

**Figure 5:**
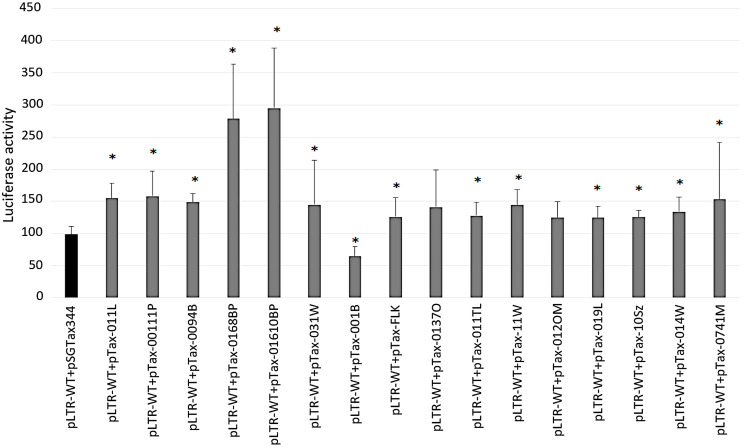
Promoter activity from the interaction of BLV Tax variants with reference strain BLV LTR. HeLa cells were cotransfected with the expression plasmids (pTax-011L, pTax-00111P, pTax-0094B, pTax-0168BP, pTax-01610BP, pTax-031W, pTax-001B, pTax-0137O, pTax-011TL, pTax-11W, pTax-012OM, pTax-019L, pTax-10Sz, pTax-014W, pTax-0741M, pTax-FLK) and the pLTR-WT. Luciferase activity generated by cotransfection of pLTR-WT, and pSGTax344 was set as a reference level of promoter activity. * Statistically significant by Mann–Whitney U Test (*p* < 0.05). Error bars shown are ± standard deviation from three independent experiments.

To determine the transactivation activity of the Tax variants, the 15 *tax* gene sequences and BLV-FLK sequence were subcloned into the pSG5 expression plasmid. HeLa cells were cotransfected with the resulting plasmids and the pLTR-WT or pGL4.74 [hRluc/TK]. Luciferase activity resulting from cotransfection of pLTR-WT and pSGTax344 was set as the reference level for promoter activity. The pTax-001B plasmid resulted in weak transactivation activity and caused a 40% decrease in promoter activity of pLTR-WT in comparison to the reference Tax plasmid (0.6-fold, SD = 0.1-fold) (Figure 5). This plasmid differed from the remaining plasmids based on a substitution at residue C(257)F located within multifunctional domain of BLV Tax (Table 2). Next, thirteen plasmids including pTax-012OM, pTax-011L, pTax-00111P, pTax-0094B, pTax-031W, pTax-0137O, pTax-011TL, pTax-11W, pTax-019L, pTax-10Sz, pTax-014W, pTax-0741M, and pTax-FLK resulted in moderate increase in promoter activity of the pLTR-WT plasmid (by 1.3- to 1.6-fold) (Table S1). The most frequent mutations noted within these plasmids were A(106)S in 13/13 plasmids, T(69)M/A in 10/13, S(140)N in 9/13, T(152)I in 9/13, T(221)S in 6/13, L(233)I/P in 6/13, and C(257)G/Y in 5/13. The last vectors, pTax-0168BP and pTax-01610BP, markedly increased pLTR-WT promoter activity (by 2.8- to 3-fold). These BLV Tax variant harbored T(69)M, L(92)F (only for pTax-01610BP), L(141)V, and S(281)P mutations (Table 2). 

### 2.7. Ability of Tax Variants to Transactivate Different BLV LTR Variants 

**Figure 6:**
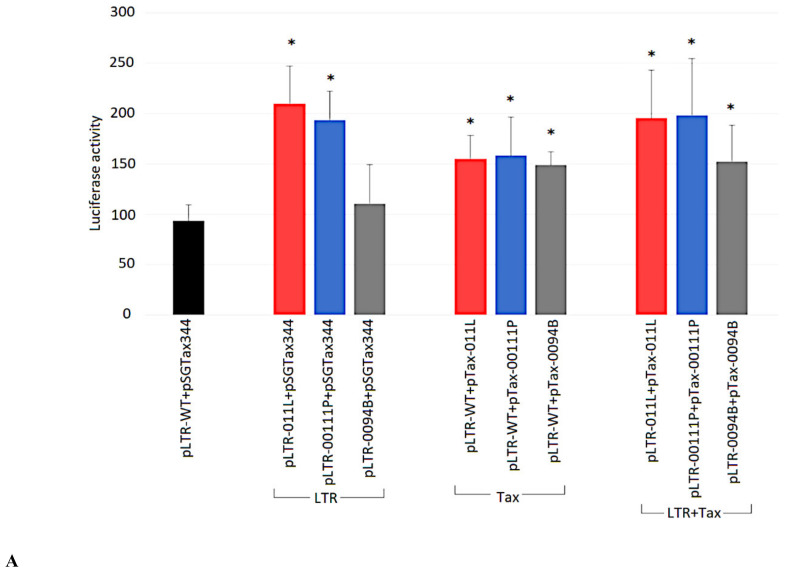

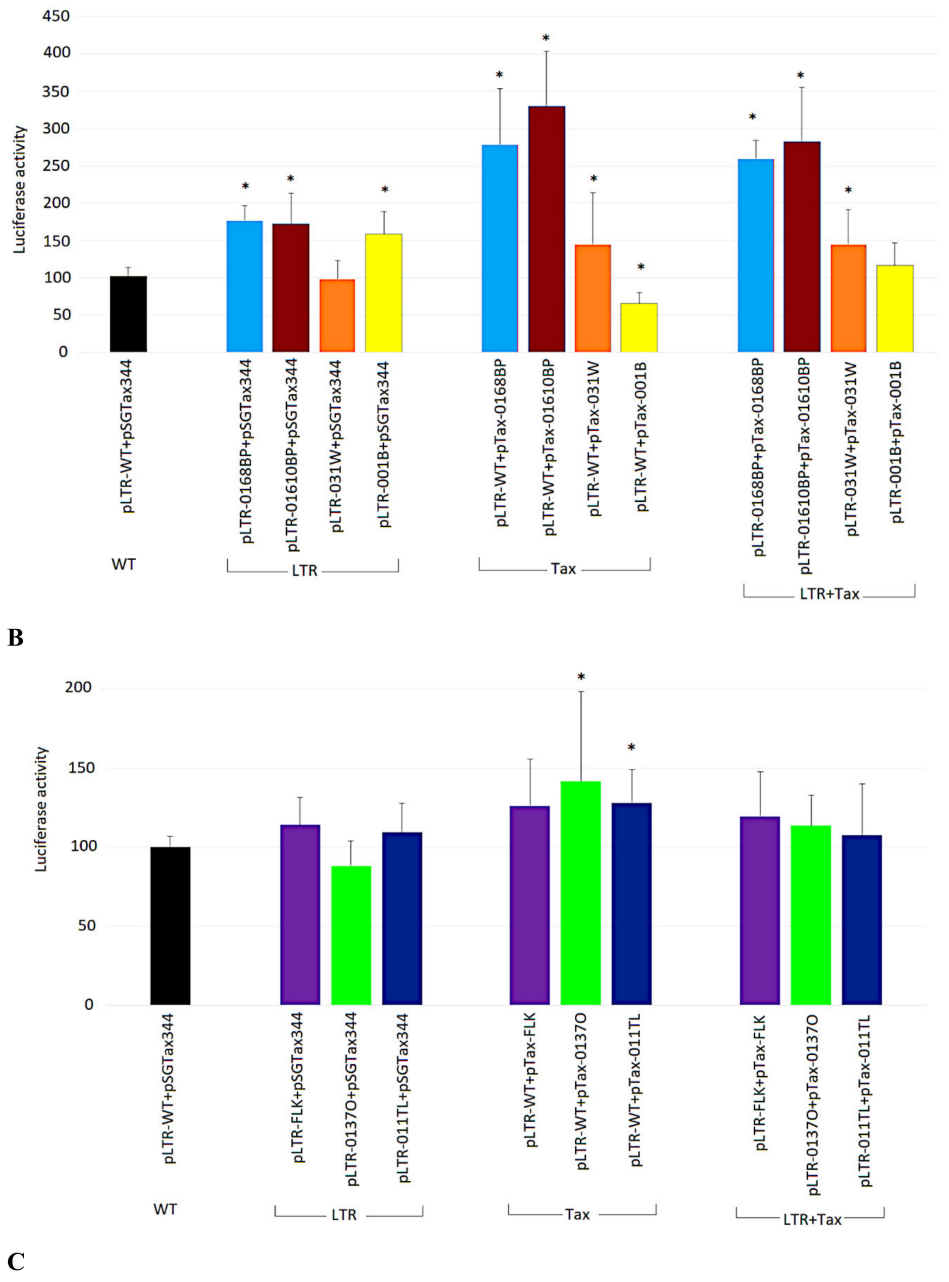

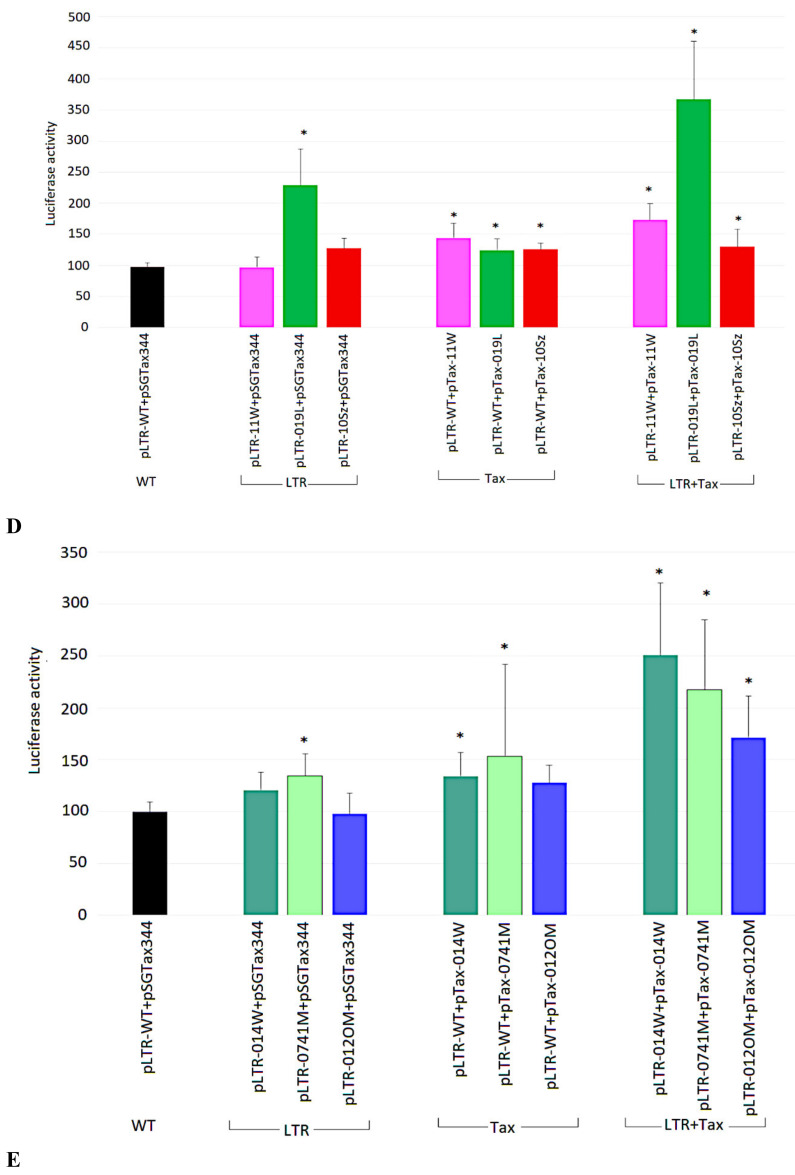

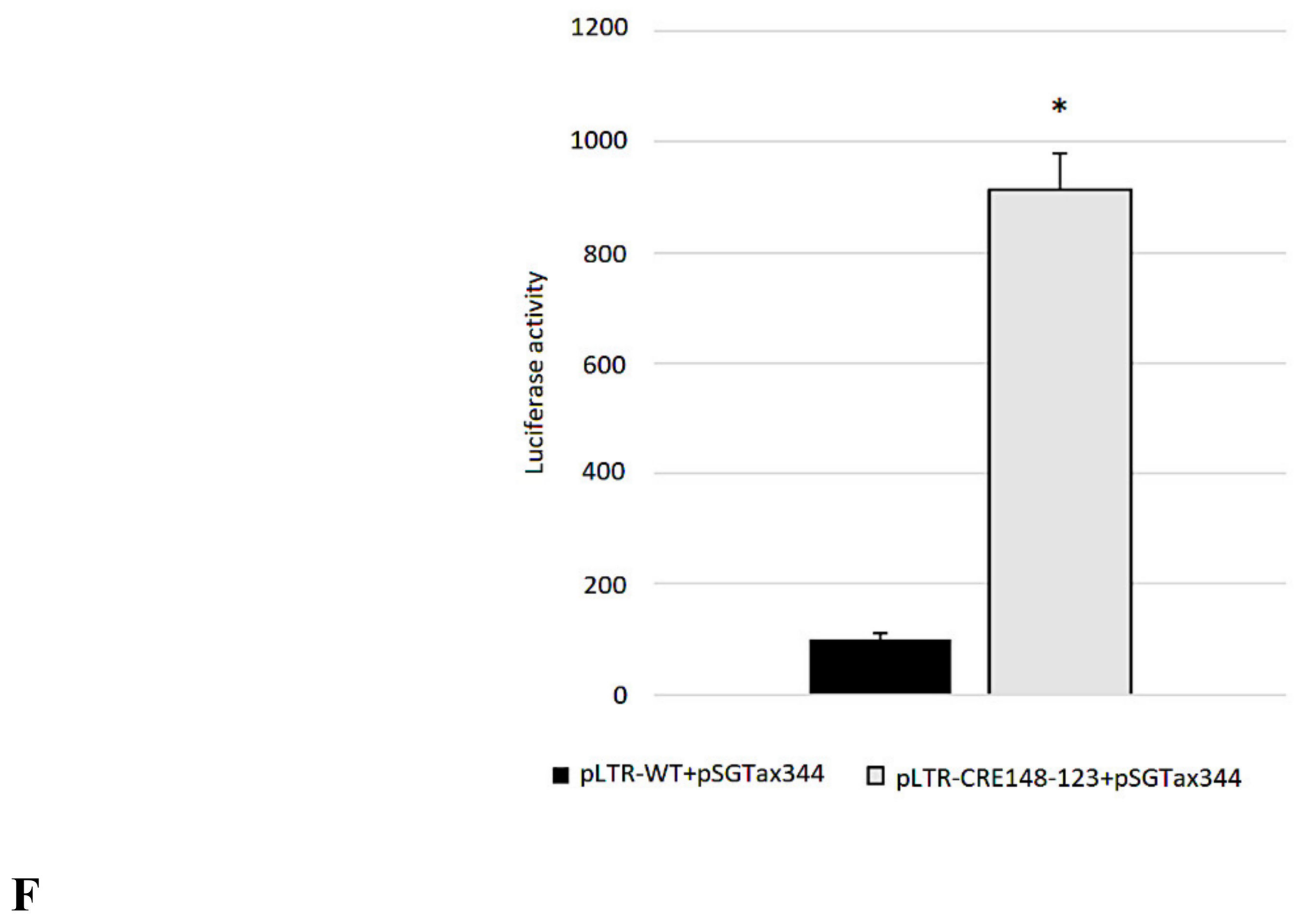
(**A**–**F**): Promoter activity from the interaction of BLV LTR variants with their corresponding Tax variants. HeLa cells were cotransfected with pLTR plasmids with the corresponding pTax plasmids (LTR + Tax). Additionally shown, is the activity from the interaction of the LTR variants with reference strain Tax (LTR) and the interaction of the Tax variants with reference LTR (Tax). (**A**) Bars marked in red, blue, and grey represent 011L, 0011P, and 0094B variants respectively—classified to genotype G4 subtypes Id-e. (**B**) Bars marked in light blue, burgundy, orange, and yellow represent 0168BP, 01610, 031W, and 001B variants, respectively, belonging to genotype G4 and subtypes G4-Ia-b. (**C**) Bars marked in violet, light green, and navy blue represent BLV-FLK, 0137O and 011TL variants respectively, assigned to genotype G1 and subtype G8-I. (**D**) Bars marked in pink, green, and red apply to 11W, 019L, and 10Sz, respectively, assigned to genotype G7-I. (**E**) Bars marked in dark green, olive, and blue represent 014W, 0741M, and 012OM variants classified to the genotype G4 and subtypes G4-II and G4-III. (**F**) Activity generated by the reference plasmids pLTR-WT + pSGTax344 (black bar) and mutant pLTR-CRE148-123 + pSGTax344 (grey bar). y-axes are different. * Statistically significant by Mann–Whitney U Test (*p* < 0.05). Error bars shown are ± standard deviation from three independent experiments.

Finally, to evaluate whether the LTR variants yield any difference in promoter activity in response to their corresponding Tax variants, the cotransfection of HeLa cells with the 15 LTR plasmids in combination with the 15 Tax expression plasmids was performed. The resultant promoter activities associated with the variants are illustrated in Figure 6A–E. 

HeLa cells cotransfected with pLTR-001B, pLTR-0137O, pLTR-011TL, or pLTR-FLK and corresponding Tax plasmids (pTax-001B, pTax-0137O, pTax-011TL, and pTax-FLK, respectively) revealed promoter activity comparable to the reference plasmid (by 1.0- to 1.2-fold) (Figure 6B,C and Table S1). Cells cotransfected with pLTR-00111P, pLTR-0094B, pLTR-031W, pLTR-11W, pLTR-10Sz, or pLTR-012OM and corresponding Tax plasmids (pTax-00111P, pTax-0094B, pTax-031W, pTax-11W, pTax-10Sz, and pTax-012OM, respectively) revealed promoter activities 1.3- to 2.0-fold higher than the pLTR-WT and pSGTax344 reference combination (Figure 6A,D,E; Table S1). 

In contrast, cotransfection of pLTR-011L, pLTR-0168BP, pLTR-01610BP, pLTR-014W, or pLTR-0741M with corresponding Tax plasmids (pTax-011L, pTax-0168BP, pTax-01610BP, pTax-014W, and pTax-0741M, respectively) resulted in promoter activity 2.1- to 3.0-fold higher than the reference combination (Figure 6A,B,E; Table S1). HeLa transfected with the pLTR-019L plasmid and pTax-019L plasmid showed promoter activity 3.7-fold higher than the reference plasmid (Figure 6D). The pLTR-CRE148-123 plasmid, harboring two consensus CRE sequences TGACGTCA in CRE1 and CRE2 motif, cotransfected with pSGTax344 reference plasmid showed promoter activity almost 10-fold higher than the reference strain pLTR-WT and pSGTax344 combination (Figure 6F). 

### 2.8. Association between BLV LTR and Tax Naturally Occurring Variants and Proviral Load 

**Figure 7:**
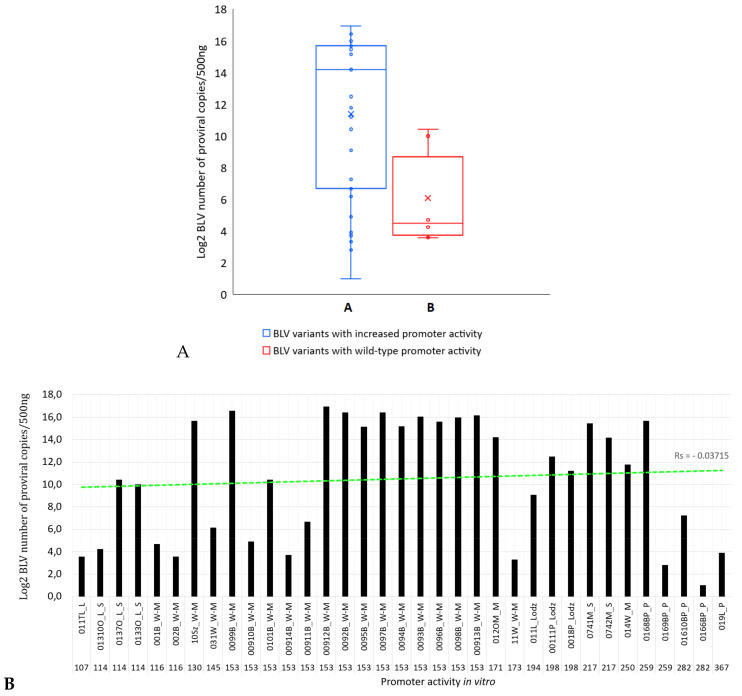
(**A**,**B**): Proviral load in thirty-five BLV isolates. (**A**) Box and whiskers plot features are as follows: central line in box is median, bottom line of box is first quartile (25%), top line of box is third quartile (75%), diamond is mean, bottom of whiskers is minimum value of copy number, top of whiskers is max copy number, and dots are raw values. Number of proviral copies is plotted on a base-2 logarithmic scale. Group A, variants with elevated transcriptional activity; group B, variants with reference strain transcriptional activity. Groups A and B were compared by using the Mann–Whitney U test, *p* < 0.03734. (**B**) Correlation between proviral copy numbers and promoter activity in vitro for the studied variants. The values of promoter activity were sorted on the x-axis from the lowest to the highest. There was no statistical correlation between the variables observed (Rs = −0.03715, *p* > 0.05). The light-green dashed line indicates the trend line.

To translate our in vitro result into clinically relevant results, we evaluated how the LTR and *tax* gene variant combinations which induced elevated promoter activity in vitro correlated with the proviral load in lymphocytes from the naturally infected cows. Due to the limited number of evaluated Tax sequences, it was only possible to analyze the proviral copy number for the select 15 LTR and Tax sequence variants and an additional 20 LTR and Tax sequences from other Polish BLV isolates that had identical LTR and Tax sequences as the selected variants. Out of the 35 LTR and *tax* sequences, 29 isolates could be grouped as variants inducing increased promoter activity (Group A) and six isolates driving promoter activity comparable with the reference strain (Group B) (Figure 7A). The BLV proviral load qPCR for the 35 lymphocyte-derived DNA samples was performed accordingly with previously published protocol [5]. The quantitative PCR assay for the samples from Group A revealed the BLV proviral copies ranged from 2 to 125,493 copies per 500 ng of genomic DNA. The number of BLV DNA copies detected in the samples from Group B ranged from 12 to 1374 copies per 500 ng of genomic DNA. To determine whether an association existed between the promoter activity and proviral load, the proviral copy numbers for 35 DNA samples from both groups were compared. Statistical analysis revealed that the variants inducing increased promoter activity possessed a significantly higher level of proviral load in comparison with the variants generating the promoter activity comparable to the reference strain 344, with *p* value <0.03734 using the Mann–Whitney U test. Correlation coefficient between proviral copy numbers and the in-vitro promoter activities was calculated (Figure 7B). There was no linear relationship observed between the proviral load and promoter activity measured in the in vitro assay (Spearman’s Rho, Rs = −0.03715, *p* (2-tailed) = 0.83219).

## 3. Discussion

In this study, samples of proviral DNA extracted from peripheral blood leukocytes (PBLs) were collected from Polish cattle naturally infected with BLV, and the sequences of the LTR region and the *tax* gene were determined in order to better understand how genomic variation is tied to BLV transactivation. Compared to reference sequences, nucleotide variations were found in enhancer and promoter sequences of the LTR. The fifteen plasmids containing unique BLV LTR sequence variants were generated and further tested using an in vitro luciferase reporter gene system in HeLa cells. 

In comparison to the reference pBLV344 strain, an increased level of promoter activity in the presence or absence of Tax was observed in 6 out of 15 LTR variants. These variants harbored changes in the middle of the U3 region, including A(−137)G, A(−135)G, and A(−134)G in κB-like site and overlapping TRE site; C(−83)T in the PU.1/Spi-B site and overlapping κB site; C(−53)A in CRE3 and G(−43)T in the TATA box; as well as mutations in R region, including CT(+191/2)ins in box C of DAS and T(+113)G in the putative MAZ site. 

Mutations in LTR regulatory sequences that recruit transcription repressors may also contribute to increased promoter activity. The E-box motif is a dual-function element that can bind different proteins acting either as activators or as repressors of transcription [51]. In the LTR, E-box elements act as repressors by binding directly to the CRE enhancers [52]. A single mutation in the E-box motif significantly increases the LTR basal promoter activity [21]. 

Brooks et al. reported that a NF-κB binding site located in the BLV LTR is involved in transcriptional activation [18,53]. The κB site is a dual-function element that can interact with repressors and compete with p65 [18,54]. For example, it is possible that A(−137)G, A(−135)G, A(−134)G, and C(−83)T mutations destroy binding sites for a repressor and enhance transcription. Consistent with Brooks et al. who showed that NF-κB activates basal and Tax-induced transcription [53], variants in our study containing changes in κB sites demonstrated increased promoter activity in presence and absence of Tax. 

Mutations G(−43)T in the TATA box and C(−53)A in the CRE3 (located immediately upstream of the TATA box) may modify the transcription initiation complex, since the transcription factors specific for these elements cooperate in their function to stabilize binding of the transcription Factor II D (TFIID) to the TATA box. The effects of mutations in the TATA box are known to be dependent on cell type, because of presence or absence of tissue specific transcription factors that can determine how the TATA box functions. The double insertion detected in CT(+191/2)ins in box C of DAS region may affect BLV promoter activity, as the three boxes A, B, and C within the DAS are the most conserved sequences, which cooperate with each other to mediate effective transcription. Our findings are consistent with the results of Kiss-Toth et al., who reported that DAS region can regulate the virus promoter independently of the Tax protein [22]. In our study, the insertion in box C increases promoter activity in presence and absence of Tax. 

It is also surprising that spontaneous mutation in the LTR can create a novel transcription factor-binding site that may play role in the promoter activation. One such element is a putative MAZ site located in the middle of the R region. Indeed, a single mutation T(+113)G found in two LTR variants significantly induced promoter activity. MAZ belongs to the Cys2His2-type zinc finger proteins (ZNFs) family and is characterized by multiple tandemly repeated zinc fingers [55]. In addition to six potential zinc fingers of the C2H2 type, MAZ contains an amino-terminal proline-rich domain and several polyalanine tracts which bind to a GA box sequence (GGGAGGG). MAZ was originally identified by its ability to bind and repress the c-MYC P2 promoter [56]. The MAZ transcription factor plays dual roles in transcription initiation and termination [57]. Interestingly, ZNFs were also shown to mediate transcriptional activation and repression of the HIV-1 LTR [58]. Unfortunately, the role of the R region in transcriptional regulation of BLV is still not clear. The functional significance of the CT(+191/2)ins and T(+113)G changes have yet to be evaluated independently, as they were identified together with other changes occurring in the U3 region regulatory elements. 

Besides 019L_P, all LTR sequence variants displaying increased promoter activity have a cytosine at position −36C. In contrast, all variants whose expression was equivalent to that of the reference provirus have a thymine at that position (−36T). These results are similar to observations by Murakami et al., who showed that the presence of T(175)C substitution in the LTR promoter region alters viral productivity by changing viral transactivation [33]. Besides 019L_P, all LTR variants that showed elevated promoter activity demonstrated strict conservation of the GRE binding site. On the other hand, the C(−65)T transition in the GRE was associated with LTR variants with reference strain promoter activity. The GRE site recognizes the glucocorticoid receptor and response to dexamethasone in presence of the Tax. It is known that the C(−65)T mutation significantly decreases basal LTR activity in reporter assay, as well as in vivo mutation of the GRE abrogates the dexamethasone response [19,59]. Finally, among the LTR sequences, no variant showed reduced promoter activity in comparison to the reference strain 344. 

Sequence analysis of the BLV Tax viral transactivator revealed the presence of missense mutations within the leucine-rich activation domain, the multifunctional domain, and within T-cell epitopes. We analyzed the activity of 15 Tax variants and the corresponding LTR sequences isolated from the same provirus. Surprisingly, increased transactivation activity (as compared with reference strain) was recorded for 14 of 15 Tax variants. Analysis of these variants showed that 7 of them had mutations in the multifunctional domain (240–265 region). The C(257)Y and C(257)G mutations occurring in three and two variants, respectively, were accompanied by other mutations located, for example, in the transactivation domain. Therefore, it was not possible to clearly show the effect of specific amino acid substitutions on the transactivating activity of the BLV Tax protein. Additionally, the 019L_P variant had a D(258)N mutation located in the multifunctional domain. In the Tajima study (2000), the clone pr2436 had a mutation in this position, i.e., D(258)G, and was characterized by increased transactivation activity. In complement, our variant 011TL_L had the S(265)G mutation, which was also observed in clone by their pr2374 [44]. Clone pr2374, like our 011TL_L variant, showed higher transactivating activity compared to infectious molecular clone pBLV-IF and λBLV-1 (accession no. K02120) [60]. However, it should be mentioned that our variants also harbored other mutations located in the transactivation domain, T-cell epitope or CD8+ CTL epitope compared to the pr2436 and pr2374 clones [44]. The L(233)P mutation of the Tax protein, which occurs in four of our study’s variants (with transactivation activity by 1.3- to 1.4-fold) was reported to be correlated with leukemogenicity of bovine leukemia virus [61]. However, its role in transactivation activity was not studied [61]. Interestingly, 7 variants that did not accumulate mutations in the Tax activation domain showed evidence of elevated transactivation activity. The mutations of these variants (T(69)M/A (7 out of 7 variants), L(141)V (2 out of 7 variants), I(186)T (1 out of 7 variants), L(233)F (1 out of 7 variants), and S(281)P (2 out of 7 variants)) are located in different sites of the BLV Tax protein. Compared to about three hundred Tax sequences deposited at GenBank, these mutations were very rare or unique. This study suggests that mutations in the 240–265 region are probably not the only ones that can impact the transactivating activity of the BLV Tax protein. In fact, in addition to the transactivation domain and the zinc finger domain, there may be other autoregulatory sequences in the Tax protein, whose location needs to be determined [46,62]. One of the tested variants of Tax, 001B, showed a reduced level of transactivation activity. This variant was characterized by five mutations, including the C(257)F transition that is located within amino acids 240–265. Unfortunately, this work did not permit the determination of whether the C(257)F change could lead to reduced Tax expression and thus affect the reduced transactivating activity of the protein in the transcription process. 

In most cases, cotransfection of HeLa cells with corresponding LTR and Tax variants resulted in increased promoter activity compared to the BLV 344 strain. Of the fifteen variants, three showed promoter activity at the level of the reference strain. In contrast, the remaining 12 variants displayed increased transactivation activity. We estimated that about 76% of the isolates from the study population could have BLV promoter activity above that of the reference 344 strain. Thus, in Polish cattle herds where BLV has not been completely eradicated, the circulating strains appear to be characterized by an increased transcriptional potential. Therefore, further studies would be desirable in order to evaluate whether circulating BLV strains in Polish cattle herds are characterized by an enhanced transcriptional activity in vivo and if this translates into a greater potential for spread than variants with a reference strain level of promoter activity. This could explain the recurrent BLV infections in already cured herds in some regions of Poland. The BLV variants with elevated levels of promoter activity were classified to genotype 4 and 7. The variants with transcriptional activity comparable to strain 344 were assigned to the genotypes 4, 8, and 1 (the BLV-FLK variant was classified to genotype 1). Therefore, we suppose that the affiliation of the BLV sequence to a particular genotype does not clearly determine its transcription properties. Nevertheless, a difference in transcriptional activity was evident at the subgroup level. According to the LTR phylogenetic tree, the variants with elevated promoter activity were assigned to the subgroup G7-I and G4-Ia, G4-Ic-e, G4-II, and G4-IIIa-c. In contrast, the variants with typical promoter activity belonged to the G8-I, G4-Ib, and G1.

The transcriptional profiles of the variants were associated with the proviral load (number of proviral DNA copies detected in qPCR). This type of association between LTR point mutations and proviral load levels have also been described in HTLV-1 asymptomatic carriers by Neto et al. [63]. They suggested that changes in domain A of the HTLV-1 TRE-1 motif resulting from a G(232)A mutation may increase HTLV-1 replication in the majority of infected individuals. Our results also suggest a possibility of such association between select mutations in LTR and Tax and the ultimate BLV proviral load. High BLV proviral loads were observed in cattle naturally infected with BLVs that carried mutations A(−137)G, A(−135)G, A(−134)G, C(−83)T, C(−53)A, G(−43)T, CT(+191/2)ins, and T(+113)G in the LTR, as well as several mutations in Tax, especially C(257)Y/G, D(258)N, T(69)M/A, L141V, and S(281)P. These isolates were also associated with elevated promoter activity. It is important to note that our study is limited by the nature of multiple SNPs within each variant, which included more than one mutation in both regulatory elements of the LTR, as well as in Tax protein.

There was a trend towards increased proviral load and increased promoter activity amongst investigated variants. However, no apparent correlation exists between the measured proviral load from BLV isolates and the respective in-vitro promoter activity in our assays, suggesting that additional mechanisms may impact proviral mRNA transcription in vivo. Mainly, transcription is dependent on the location of the provirus in the bovine genome (an insertion site) and the chromatin structure, which can impose significant obstacles in transcription mediated by RNA polymerase II [64,65,66,67]. Histone modification, chromatin remodeling, histone variant incorporation, and histone eviction affects binding of transcription factors to the LTR promoter as well as the initiation and elongation steps of transcription [68,69,70]. This means that even an efficient promoter (based on transcription binding sites) may be silenced due to the insertion site and the chromatin context. For example, Murakami et al. showed that the spontaneous mutation at a single nucleotide 175 in the TATA box-binding protein site modified viral transactivation [33]. However, there was no significant difference in proviral load between strains harboring either T or C at 175 position [33,71]. 

Conversely, robust viral expression means potential exposure to the immune system. Merezak et al. showed that reconstitution of a CRE consensus in the triplicate motif of the imperfectly conserved TxRE sequences has a drastic effect in a transfection assay, leading to 20-fold induction in basal transcriptional activity in lymphocytes [15]. This was associated with an absolute level of CRE3x-induced transcription and the highest complex formation efficiency between CREB/ATF factors and TxRE. The reconstitution of a perfect CRE consensus induced also a drastic reduction in BLV propagation and restricted the proviral load in vivo, likely due to the host immune response [15,72]. In our study, besides single C(−53)A mutation in CRE3 in the 01610BP_P variant (which did not restore the CRE consensus sequence TGACGTCA), no mutation in CRE sequences was noted in the examined variants, indicating that the main strategy of the repression of viral genes expression was intact. The in-vitro analysis of the promoter strengths of the examined BLV LTR variants demonstrated that the promoters of the three variants had similar activity to the reference strain 344, while the strength of the remaining 12 promoters was no more than 3.7-fold higher and had a modest impact on transactivation. 

The 5′-LTR contains transcription factor binding sites that mediate promoter activity upon regulation by a series of signaling pathways. In fact, LTR promoters contain numerous *cis*-regulatory elements, which determine the maximal rate of viral transcription initiation. However, the cell type and its differentiation state with respect to a variety of cell activation signals may lead to substantial variations in transcriptional activity of an LTR. All these variables generate a remarkably wide range of levels for viral gene expression. Here, we reported measurements of LTR-driven gene expression upon transfection of epithelial HeLa cells in the presence or absence of Tax protein. Therefore, the presence of cell-type specific combination of TFs in HeLa cells may generate distinct transcriptional activity in comparison to the other cell lines. 

Further studies based on reverse genetics are necessary to determine the association between transactivation and proviral burden in infected cells.

The detected variability in the BLV LTR sequences—and consequently, the different promoter activity—demonstrates that the transcriptional regulation for each BLV isolate is not identical to that of reference type strain 344, and different trends in the evolution of individual LTRs take place. However, available data do not allow for speculation of whether the differences observed in the promoter sequence and activity of different BLV isolates represent adaptive changes due to the host’s health status or environmental conditions. 

## 4. Materials and Methods 

### 4.1. Sample Collection and Preparation 

Blood samples from cattle naturally infected BLV were selected from collections at local diagnostic laboratories as part of the EBL monitoring program between 2013 and 2018 and sent to the National Veterinary Research Institute for secondary study. A total of one hundred and six blood samples were collected from cattle from 51 herds within different regions of Poland, where recurrent BLV infections were noted (Table S2). BLV infection status was determined serologically using a commercially available ELISA kit: IDEXX Leukosis Blocking and Leukosis Serum X2 (IDEXX, Switzerland) according to the manufacturer’s instructions. The animals used in this study included BLV-seropositive cattle showing no persistent lymphocytosis or clinical signs indicating tumor form of EBL. The peripheral blood leukocytes (PBLs) were isolated by centrifugation at 1500× *g* for 25 min and erythrocytes were hemolyzed by osmotic shock with H_2_O and 4.5% NaCl, as previously described [36]. Genomic DNA was extracted using a DNeasy Blood & Tissue Kit (Qiagen, Valencia, CA, USA) following the manufacturer’s instructions, and the samples were stored at −20 °C until use. 

### 4.2. Amplification and Sequencing of LTR and Tax Gene 

The full-length LTR was amplified by overlap extension PCR using oligonucleotide primers, as previously described [36]. Three independent PCR amplifications were performed using KAPA Taq polymerase, amplicons were sequenced, and consensus sequence was generated to ensure polymerase fidelity. The *tax* gene was amplified by nested PCR amplification using two sets of primers described in Table S3. First round of amplification was performed using PrimeSTAR GXL DNA Polymerase and Prime STAR GXL buffer (Takara Bio, Shiga, Japan). Thermal cycling conditions were as follows: 2 min at 98 °C, 38 cycles (15 s at 98 °C, 25 s at 56 °C, 2 min 30 s at 68 °C), and 10 min at 72 °C. The nested PCR was performed using Q5 High-Fidelity DNA Polymerase and Q5 Reaction Buffer (New England BioLabs, MA, USA). Thermal protocol for nested PCR were as follows: 30 s at 98 °C, 35 cycles (10 s at 98 °C, 25 s at 46 °C, 1 min at 72 °C), and 10 min at 72 °C. PCR products were separated and analyzed by electrophoresis on 1.5% agarose gel containing SimplySafe (EURx, Gdańsk, Poland), diluted 1:10,000 in 1x TAE buffer. The PCR products were purified using a NucleoSpin Extract II Kit (Marcherey Nagel GmbH & Co KG, Germany) and sequenced in Genomed SA Company (Warsaw, Poland) using a 3730xl DNA Analyzer (Applied Biosystems, Foster City, CA, USA) and a Big Dye Terminator v3.1 Cycle Sequencing Kit. Raw sequence data were proofread in Geneious Prime 2019.0.3 (Biomatters Ltd., Auckland, New Zealand). Proviral sequences obtained from the individual animals did not show any variation. The data were deposited in the GenBank database under accession numbers included in Table S2. 

### 4.3. Sequence Data Analysis 

The LTR and *tax* gene sequences generated from BLV field isolates were aligned with subclone pBLV913 (BLV-FLK) and reference BLV provirus (strain 344) sequences using ClustalW algorithm implemented in Geneious Prime 2019.0.3. For the LTR alignment, the HKY85 substitution model was applied in Geneious Prime to infer a phylogenetic tree according to Bayesian method. For the Tax protein sequences, the Bayesian inference under the Poisson model was calculated [73]. To analyze the transcription factor binding site (TFBS) modifications related to specific mutations in LTR, the Geneious Prime plugin based on the EMBOSS 6.5.7 tool tfscan was used [74]. Out of the one hundred and six samples, forty-seven had previously reported LTR sequences [36].

### 4.4. Construction of Plasmids

#### 4.4.1. PCR Amplification of LTR and *tax* Sequences 

To construct luciferase-based reporter plasmids containing observed mutations in LTR regulatory elements, an overlap extension PCR was used to amplify each particular LTR variant. The first round of PCR allowed for the amplification of two sequences encompassing the LTR: a 5’-end fragment of 979 bp and 3’-end fragment of 571 bp were generated using Q5 High-Fidelity DNA Polymerase and Q5 Reaction Buffer (New England BioLabs, MA, USA) at the following thermal conditions: 30 s at 98 °C, 40 cycles (10 s at 98 °C, 45 s at 65 °C (5’-end fragment) or 52 °C (3’-end fragment), 1 min at 72 °C), and 7 min at 72 °C. The resulting amplicons were subjected to a second PCR using P8169 and P520 oligonucleotides described in Table S3 and Q5 High-Fidelity DNA Polymerase (New England BioLabs, MA, USA), as described above. Thermal conditions were as follows: 30 s at 98 °C, 38 cycles (10 s at 98 °C, 50 s at 64 °C, 1 min 15 s at 72 °C), and 9 min at 72 °C. To construct Tax expression plasmids, the *tax* gene was amplified by nested PCR using the oligonucleotide primers described in Table S3. First round PCR was performed as described in Section 2.2. Nested PCR was carried out using Q5 High-Fidelity DNA Polymerase with the following thermal conditions: 30 s at 98 °C, 35 cycles (10 s at 98 °C, 25 s at 58 °C, 1 min at 72 °C), and 10 min at 72 °C.

#### 4.4.2. Cloning of LTR and Tax Sequences in Plasmid Vectors

The amplicons representing LTR sequences and *tax* gene were purified on NucleoSpin Gel and PCR Clean-up columns (Macherey-Nagel GmbH & Co KG, Germany) and cloned into vector pMiniT 2.0 using the NEB PCR Cloning Kit with competent cells (New England BioLabs, MA, USA), according to the manufacturer’s protocol. Next, the LTR inserts were subcloned in the NheI-HindIII sites of pGL4.11[*luc2P*] (Promega, Madison, WI, USA) to construct pLTR-011L, pLTR-00111P, pLTR-0094B, pLTR-0168BP, pLTR-01610BP, pLTR-031W, pLTR-001B, pLTR-0137O, pLTR-011TL, pLTR-11W, pLTR-012OM, pLTR-019L, pLTR-10Sz, pLTR-014W, pLTR-0741M, and pLTR-FLK plasmids. Then, the NEB 10-beta Competent *E. coli* (DH10B derivative) were used for transformations and maintenance of plasmid DNA. To verify the insert upstream of the luciferase reporter gene *luc2P,* the pGL4.11 recombinant was sequenced using a RVprimer3. The wild-type BLV strain 344, which was originally isolated as a provirus from a BLV-induced tumor [75] was used as reference strain in this study. BLV 344 provirus is available in the plasmid pBLV344H, as described in Willems et al. 1993 [43], and is deposited under the Budapest Treaty with the Belgian Coordinated Collections of Microorganisms BCCM/LMBP Collection under accession number LMBP 8165. It is the most well-characterized and widely used in BLV sequence in a wide-range studies [15,72,76,77]. What is important for this research, is that the BLV strain 344 was classified to genotype 4 and characterized by high sequence similarity to the new strains used in this study. Therefore, the vectors pLTR-WT and pSGTax344 that represent strain 344 were used in this study as controls. Additionally, vector pLTR-CRE148-123, described in detail elsewhere, was used as positive control [15]. The *tax* inserts were subcloned into the pSG5 vector. The ClaI–SacI fragment of reference strain *tax* gene was excised from plasmid pSGTax344 and was replaced by *tax* gene variants. Restriction analysis and sequencing with primers P7806 and P1101 were performed to verify the integrity of the resulting expression plasmids named as follows: pTax-011L, pTax-00111P, pTax-0094B, pTax-0168BP, pTax-01610BP, pTax-031W, pTax-001B, pTax-0137O, pTax-011TL, pTax-11W, pTax-012OM, pTax-019L, pTax-10Sz, pTax-014W, pTax-0741M, and pTax-FLK. Finally, bacterial cultures for pLTRs and pTax plasmids preparation were grown for 14 h in Luria Bertani (LB) medium supplemented with ampicillin (100 µg/ml) and purified using QIAGEN Plasmid Maxi Kit according to manufacturer’s protocol. 

### 4.5. Cell Culture 

HeLa cells were maintained in Dulbecco’s modified Eagle medium (DMEM) with 4.5 g/L Glucose, with L-Glutamine (Lonza BioWhittaker, Verviers, Belgium) supplemented with 10% heat-inactivated fetal calf serum (Gibco, Thermo Fisher, USA), and 1% penicillin-streptomycin mixture (Lonza BioWhittaker, Verviers, Belgium) and were cultured at 37 °C in an incubator with 5% CO_2_. 

### 4.6. Luciferase Reporter Gene Assay

The HeLa cells were transfected with pGL4.11 plasmids containing LTR sequences, pSG5 plasmids with *tax* gene inserts, and pGL4.74[hRluc/TK] encoding Renilla (Promega, Madison, WI, USA). One day before transfection, the cells were cultured at density of 5 × 10^4^ cells per well (24-well dish, Nunc, Thermo Fisher Scientific, The Netherlands). The day of transfection, transfection mix was prepared by mixing 1µg pLTR, 100 ng pSGTax or pSG and 6 ng Renilla in serum-free DMEM. Then, 2 µl of *Trans*IT-LT1 Transfection Reagent (Mirus Corporation, Madison, WI, USA) per 1 µg of plasmid DNA was added to the transfection mix, incubated at room temperature for 15 min and added to the cells in triplicate wells. 24 h after transfection, the cells were washed with phosphate-buffered saline (PBS) and cell extracts were prepared with lysis buffer (Dual-Luciferase Reporter Assay System, Promega, Madison, WI, USA). The luciferase activity was measured using a Dual-Luciferase Reporter Assay kit (Promega) and a Luminoskan Ascent luminometer (Thermo Labsystems, Helsinki, Finland). Luciferase assay results were normalized as follows: Normalized activity = Firefly Luciferase activity/Renilla Luciferase activity. All assays were carried out in triplicate.

### 4.7. Statistical Analysis

Differences in luciferase activities were analyzed by Mann–Whitney U Test. As a measure of variation in luciferase activity, the coefficient of variation (CV) was used. CV = (σ/mean luciferase activity level) × 100. The difference in luciferase activity between LTR variants and the same variants cotransfected with Tax was assessed using Wilcoxon signed-ranks test. All the statistical analyses were performed using STATISTICA version 10 (StatSoft, Tulsa, OK, USA), where *p* value of <0.05 was considered to be significant.

### 4.8. Quantification of Proviral DNA by Real Time PCR

BLV qPCR was performed according to previously published methods [78]. Briefly, genomic DNA was amplified by TaqMan PCR with primers for *pol* gene (5′-CCTCAATTCCCTTTAAACTA-3′ and 5′-GTACCGGGAAGACTGGATTA-3′) and probe 6FAM GAACGCCTCCAGGCCCTTCABHQ1 in Rotor-Gene Q cycler using QuantiTect Multiplex PCR NoROX master mix (Qiagen AG GmbH, Germany) according to the protocol: 15 min denaturation at 95 °C followed by 45 cycles (60 s at 94 °C and 60 s at 60 °C). Tenfold dilutions of the pBLV1 plasmid were made from 1 × 10^6^ copies per μL to 100 copies per μL and were used as a standard to estimate BLV copy numbers.

## 5. Conclusions

In this paper, we have characterized mutations in BLV Tax and LTR regulatory elements that are present in Polish BLV-infected cattle herds. These mutations are associated with a range of viral transactivation activity. Slight increase in promoter activity was associated with an increased proviral load in peripheral blood leukocytes of infected cows. Therefore, functional nucleotide mutations found in the LTR and *tax* gene of BLV-infected cows may impose an increase in overall proviral load and thus, impact the transmissibility of BLV.

## Figures and Tables

**Table 1:** Variations in fifteen BLV LTR sequences from cattle isolates. All the indicated variations are compared with reference strain 344 sequence shown in Figure 1.

		Nucleotide Changes within LTR																			
Subregion				U3									R								U5
Regulatory Site		NF-κB-Like Protein Site		κB/ TRE2	PU.1/ Spi-B	GRE	CRE3	TATA Box		TATA Box-Binding Protein Site		CAP Site	Putative MAZ	DAS Box A	DAS		DAS Box C				IRF
Position		−137	−135	−134	−83	−65	−53	−43	−41	−37	−36	−11	+113	+150	+182	+183	+188/9	+190	+191/2	+211	+256
Sequence variants	p344	A	A	A	C	C	C	G	T	A	C	T	T	A	T	C	TC	T	--	T	T
	011L	G	G	.	.	.	.	T	.	T	.	.	.	.	.	.	.	.	--	.	.
	00111P	G	G	.	.	.	.	T	.	T	.	.	.	.	.	.	.	.	--	C	.
	0094B	.	.	.	.	.	.	.	.	T	.	.	.	.	.	.	.	C	--	.	.
	0168BP	.	.	.	.	.	.	.	.	.	.	.	G	.	.	.	.	.	--	.	.
	01610BP	.	.	.	.	.	A	.	.	.	.	.	G	.	.	.	.	.	--	.	.
	031W	.	.	.	.	.	.	.	.	T	.	.	.	.	.	.	.	.	--	.	.
	001B	.	.	.	T	.	.	.	.	T	.	.	.	.	.	.	.	.	CT	.	.
	0137O	.	.	.	.	.	.	.	.	T	T	.	.	.	.	.	CT	.	--	.	C
	011TL	.	.	.	.	.	.	.	.	T	T	.	.	.	.	.	CT	.	--	.	.
	11W	.	.	.	.	T	.	.	A	T	T	.	.	G	.	.	.	.	--	.	.
	012OM	.	.	.	.	T	.	.	.	T	T	.	.	.	C	.	.	.	--	.	.
	019L	.	.	G	.	T	.	.	A	T	T	.	.	G	.	.	.	.	--	.	.
	10Sz	.	.	.	.	T	.	.	A	T	T	.	.	G	.	T	.	.	--	.	.
	014W	.	.	.	.	T	.	.	.	T	T	.	.	.	.	.	.	.	--	.	.
	0741M	.	.	.	.	T	.	.	.	T	T	del	.	.	.	.	.	.	--	.	.
	FLK	.	.	.	.	T	.	.	.	T	T	.	.	.	.	.	.	.	--	.	.

Abbreviations: del, deletion; --, insertion CT at +191/2 position for 001B isolate; NF-κB-like protein site, nuclear factor-like protein binding site; PU.1 and Spi-1, erythroblast transformation specific family members (Ets); TRE2, Tax Responsive Element 2; GRE, glucocorticoid response element; CRE3, cAMP-response element 3; CAP, Catabolite Activator Protein; DAS, downstream activator sequence; IRF, interferon regulatory factor binding site.

**Table 2:** Variations in BLV Tax amino acid sequences from cattle isolates.

Main/Epitope		Position	Tax Variants																
			p344	011L	00111P	0094B	0168BP	01610BP	031W	001B	0137O	011TL	11W	012OM	019L	10Sz	014W	0741M	BLV- FLK
Changes within Tax	-	56	T	.	.	.	.	.	.	.	.	.	.	.	.	.	.	.	N
	-	64	F	.	.	.	.	.	.	.	.	.	.	.	.	.	.	Y	C
	-	69	T	M	M	M	M	M	M	.	A	A	A	.	A	A	M	.	.
	-	80	R	.	.	.	.	.	.	H	.	.	.	.	.	H	.	.	.
	-	92	P	.	.	.	.	.	.	.	.	.	.	.	.	.	.	L	.
	-	93	L	.	.	.	.	F	.	.	.	.	.	.	.	.	.	.	.
	-	95	A	.	.	.	.	.	.	.	.	.	T	.	T	T	.	.	.
	-	97	R	.	.	.	.	.	.	.	.	.	.	.	.	.	H	.	.
	-	110	Q	.	.	.	.	.	.	.	R	R	.	.	.	.	.	.	R
	T-cell epitope	130	I	.	.	.	.	.	.	.	.	.	.	.	.	.	.	L	.
		140	S	.	.	.	.	.	.	.	N	N	N	N	N	N	N	N	N
		141	L	.	.	.	V	V	.	.	.	.	.	.	.	.	.	.	.
		142	V	.	.	.	.	.	.	.	.	.	.	A	.	.	.	.	.
		148	G	.	.	.	.	.	.	.	.	.	.	.	.	.	.	.	R
	CTL epitope	152	T	.	.	.	.	.	.	.	I	I	I	I	I	I	I	I	I
	Leucine-rich activation domain/CTL epitopes	164	P	.	.	.	.	.	.	.	.	.	.	.	.	.	.	.	S
		173	L	.	.	.	.	.	.	.	.	.	P	.	P	P	.	.	.
		183	R	.	.	.	.	.	.	.	.	.	.	.	.	.	.	K	.
		186	I	.	.	.	.	.	.	.	T	T	.	.	.	.	.	.	.
	-	214	I	.	.	.	.	.	.	.	.	.	.	M	.	.	.	.	.
	-	221	T	.	.	.	.	.	.	.	S	S	S	.	S	S	.	.	S
	-	222	N	.	.	.	.	.	.	.	.	.	D	.	D	D	.	.	.
	-	226	L	.	.	.	.	.	.	.	P	P	.	.	.	.	.	.	.
	-	233	L	.	.	.	.	.	F	.	I	I	P	P	P	P	.	.	.
	MD	257	C	.	.	.	.	.	.	F	.	.	G	Y	.	G	Y	Y	.
		258	D	.	.	.	.	.	.	.	.	.	.	.	N	.	.	.	.
	MD/B-cell epitope	265	S	.	.	.	.	.	.	.	.	G	.	.	.	.	.	.	.
	-	281	S	.	.	.	P	P	.	.	.	.	P	.	P	P	.	.	.
	-	287	G	.	.	.	.	.	.	.	.	.	.	.	.	.	.	.	R
	-	288	L	.	.	.	.	.	.	.	.	.	.	.	.	.	.	.	I

Indicated variations are compared with reference strain 344 sequence shown in Figure 1. Single letter codes for the 20 amino acids, according IUPAC, have been used. Abbreviations: MD, multifunctional domain; CTL, Cytotoxic T lymphocytes.

## Supplementary Materials

The following are available online at https://www.mdpi.com/2076-0817/9/10/836/s1, **Figure S1:** Alignment of LTR region nucleotide sequences of 106 Polish BLV strains. Divergence from the reference strain 344 sequence are indicated. Distribution of corresponding regulatory elements along the LTR are indicated in the header. Horizontal dashed lines above the nucleotide sequence alignment indicate the TxRE, Ebox, TATA box, κB, GRE, PU.1/Spi-B, CAT, CAP site, DAS, and IRF. Double line below the strain 344 sequence indicate TATA Box-binding protein site; a single line below the strain 344 sequence indicate the κB-like site; boxes from A to C within DAS and USF. The most frequent changes located in the regulatory elements in the U3 and R region were marked in blue. The changes that created a putative MAZ site were marked in yellow. **Figure S2:** Phylogenetic analysis of LTR sequences. Phylogenetic relationship of a 531-bp region of the 106 LTR nucleotide sequences, BLV-FLK sequence and strain 344 (pBLV344) (*n* = 108), as inferred by Bayesian analysis. Numbers at nodes indicate posterior probabilities of sampling the node among 10,000 trees. Subtype terminology is based on that of Pluta et al. [36]. LTR sequence variants selected for further study are indicated at the right by red squares. **Figure S3:** Alignment of the translated amino acid sequences of Tax protein from forty-three Polish BLV strains. The points at which translated proviral Tax sequences differ from the reference are indicated below the strain 344 sequence. Functionally important regions include the following: Zn finger domain, phosphorylation sites, T-cell epitopes, B-cell epitope, Leucine-rich activation domain, Multifunctional domain, and CD8+ CTL epitopes which are identified with dashes lines. GenBank accession numbers for 43 new *tax* gene sequences are incrementally MT723750 to MT723792. **Figure S4:** Phylogenetic analysis of Tax amino acid sequences. Phylogenetic relationship of a 310-aa fragment of 43 *tax* gene amino acid sequences, BLV-FLK, and strain 344 (pBLV344) (*n* = 45), as inferred by Bayesian analysis. Numbers at nodes indicate posterior probabilities of sampling the node among 10,000 trees. Tax variants selected for further analysis are indicated at the right by red squares. **Table S1:** Differences in luciferase activity level between the 344 strain and different LTR and Tax variants. **Table S2:** Identity and origin of the sequences analyzed in the study. **Table S3:** Sequences of primers used to amplify, sequence, and clone the BLV LTR and *tax* regions.
