# Supplementary material for: Effects of Naturally Occurring Mutations in Bovine Leukemia Virus 5′-LTR and Tax Gene on Viral Transcriptional Activity
Source: Pathogens. 2020 Oct 13;9(10):836. doi: 10.3390/pathogens9100836 (PMC7656303; doi:10.3390/pathogens9100836)
Supplement: Supplementary file 1 [file pathogens-09-00836-s001.zip › Figures.rtf]

                                                                                     NF-kB-like  
                                                                                     protein site
                                                              TRE1   CRE1     Ebox1         TRE2  CRE2   Ebox2     kB-site              CAT  PU.1/Spi-B         GRE
 U3                                                          -----------------------      ---------------------- -------------------------------------          --        
pBLV344      TGTATGAAAG ATCATGCCGG CCTAGGCGCC GCCACCGCCC CGTAAACCAG ACAGAGACGT CAGCTGCCAG AAAAGCTGGT GACGGCAGCT GGTGGCTAGA ATCCCCGTAC CTCCCCAACT TCCCCTTTCC CGAAAAATCC  
0258G_W-M    .......... .......T.. .......... .......... .......... .......... .......... .......... .......... .......... .......... .......... .......... ..........  
0253G_W-M    .......... .......T.. .......... .......... .......... .......... .......... .......... .......... .......... .......... .......... .......C.. ..........  
0257G_W-M    .......... .......T.. .......... .......... .......... .......... .......... .......... .......... .......... .......... .......... .......... ..........  
0252G_W-M    .......... .......T.. .......... .......... .......... .......... .......... .......... .......... .......... .......... .......... .......... ..........  
042AK_K-P    .......... .......T.. .......... .......... .......... .......... .......... .......... .......... .......... .......... .......... .......... ..........  
0242K_W-M    .......... .......T.. .......... .......... .......... .......... .......... .......... .......... .......... .......... .......... .......... ..........  
0256G_W-M    .......... .......T.. .......... .......... .......... .......... .......... .......... .......... .......... .......... .......... .......... ..........  
0251G_W-M    .......... .......T.. .......... .......... .......... .......... .......... .......... .......... .......... .......... .......... .......... ..........  
0255G_W-M    .......... .......T.. .......... .......... .......... .......... .......... .......... .......... .......... .......... .......... .......... ..........  
0741M_S      .......... .......T.. .......... .......... .......... .......... .......... .......... .......... .......... .......... .......... .......... ..........  
0133Z_S      .......... .......T.. .......... .......... .......... .......... .......... .......... .......... .......... .......... .......... .......... ..........  
0131Z_S      .......... .......T.. .......... .......... .......... .......... .......... .......... .......... .......... .......... .......... .......... ..........  
0742M_S      .......... .......T.. .......... .......... .......... .......... .......... .......... .......... .......... .......... .......... .......... ..........  
026Z_S       .......... .......T.. .......... .......... .......... .......... .......... .......... .......... .......... .......... .......... .......... ..........  
020B_S       .......... .......T.. .......... .......... .......... .......... .......... .......... .......... .......... .......... .......... .......... ..........  
0132Z_S      .......... .......T.. .......... .......... .......... .......... .......... .......... .......... .......... .......... .......... .......... ..........  
053K_S       .......... .......T.. .......... .......... .......... .......... .......... .......... .......... .......... .......... .......... .......... ..........  
0371B_W-M    .......... .......... .......... .......... .......... .......... .......... .......... .......... .......... .......... .......... .......... ..........  
0374B_W-M    .......... .........A .......... .......... .......... .......... .......... .......... .......... .......... .......... .......... .......... ..........  
0378B_W-M    .......... .......... .......... .......... .......... .......... .......... .......... .......... .......... .......... .......... .......... ..........  
3208M_W-M    .....A.... .........A .......... .......... .......... .......... .......... .......... .......... .......... .......... .......... .......... ..........  
3175aM_W-M   .....A.... .........A .......... .......... .......... .......... .......... .......... .......... .......... .......... .......... .......... ..........  
3205M_W-M    .....A.... .........A .......... .......... .......... .......... .......... .......... .......... .......... .......... .......... .......... ..........  
3176aM_W-M   .....A.... .........A .......... .......... .......... .......... .......... .......... .......... .......... .......... .......... .......... ..........  
012OM_M      .......... .........A .......... .......... .......... .......... .......... .......... .......... .......... .......... .......... .......... ..........  
047P_Lodz    .......... .......... .......... .......... .......... .......... .......... .......... .......... .......... .......... .......... .......... ..........  
0222GD_K-P   .......G.. .......... .......... .......... .......... .......... .......... ........A. .......... .......... .......... .......... .......... ..........  
0221AGD_K-P  .......G.. .......... .......... .......... .......... .......... .......... ........A. .......... .......... .......... .......... .......... ..........  
0221GD_K-P   .......... .......... .......... .......... .......... .......... .......... ........A. .......... .......... .......... .......... .......... ..........  
014W_M       .....A.... .........A .......... .......... .......... .......... .......... .......... .......... .......... .......... .......... .......... ..........  
014NN_K-P    .......... .......... .......... .......... .......... .......... .......... .......... .......... .......... .......... .......... .......... ..........  
0166BP_P     .......... .......... .......... .......... .......... .......... .......... .......... .......... .......... .......... .......... .......... ..........  
01610BP_P    .......... .........A .......... .......... .......... .......... .......... .......... .......... .......... .......... .......... .......... ..........  
0168BP_P     .......... .........A .......... .......... .......... .......... .......... .......... .......... .......... .......... .......... .......... ..........  
0169BP_P     .......... .......... .......... .......... .......... .......... .......... .......... .......... .......... .......... .......... .......... ..........  
022WM_M      .......... .......... .....A.... .......... .......... .......... .......... .......... .......... .......... .......... .......... .......... ..........  
0167BP_P     .......... .......... .......... .......... .......... .......... .......... .......... .......... .......... .......... .......... .......... ..........  
4W_W-M       .......... .......... .......... .......... ..C....... .......... .......... .......... .......... .......... .......... .......... .......... ..........  
0071B_W-M    .......... .......... .......... .......... ..C....... .......... .......... .......... .......... .......... .......... .......... .......... ..........  
015W_W-M     .......... .......... .......... .......... ..C....... .......... .......... .......... .......... .......... .......... .......... .......... ..........  
009B_W-M     .......... .......... .......... .......... ..C....... .......... .......... .......... .......... .......... .......... .......... .......... ..........  
0362B_W-M    .......... .G........ .......... .......... T.C....... .......... .......... .......... .......... .......... .......... .......... .......... ..........  
035S_P       .......... .......... A........A .......... .......... .......... .......... .......... .......... .......... .......... .......... .......... ..........  
006B_W-M     .A........ .......... A...CTA..A .......... ..C....... .......... .......... .......... .......... .......... .......C.. .......... .......... ..........  
0244K_W-M    .......... .......... .......... .......... .......... .......... .......... .......... .......... .......... .......... .......... .......... ..........  
015P_G_P     .A........ .......... .......... .......... .......... .......... .......... .......... .......... .......... .......... .......... .......... ..........  
031W_W-M     .......... .......... .......... .......... .......... .......... .......... .......... .......... .......... .......... .......... .......... ..........  
017B_W-M     .......... .......... .......... .......... .......... .......... .......... .......... .......... .......... .......... .......... .......... ..........  
038W_W-M     .......... .......... .......... .......... .......... .......... .......... .......... .......... .......... .......... .......... .......... ..........  
00111P_Lodz  .......... ......A... .......... .......... .......... .......... .......... G.G....... .......... .......... .......... .......... .......... ..........  
0018P_Lodz   .......... ......A... .......... .......... .......... .......... .......... G.G....... .......... .......... .......... .......... .......... ..........  
0057P_Lodz   .......... ......A... .......... .......... .......... .......... .......... G.G....... .......... .......... .......... .......... .......... ..........  
011L_Lodz    .......... ......A... .......... .......... .......... .......... .......... G.G....... .......... .......... .......... .......... .......... ..........  
0378B17_W-M  .......... ......A... .......... .......... .......... .......... .......... G.G....... .......... .......... .......... .......... .......... ..........  
001B_W-M     .......... .......... .......... .......... .......... .......... .......... .......... .......... .......... .......... .......... ........T. ..........  
002B_W-M     .......... .......... .......... .......... .......... .......... .......... .......... .......... .......... .......... .......... ........T. ..........  
0072B_W-M    .......... .........A ...G...... .......... .......... .......... .......... .......... .......... .......... .......... .......... .......... ..........  
0102B_W-M    .......... .......... ...G...... .......... .......... .......... .......... .......... .......... .......... .......... .......... .......... ..........  
030O_W-M     .......... .........A .......... .......... .......... .......... .......... .......... .......... .......... .......... .......... .......... ..........  
0097B_W-M    .......... .......... .......... .......... .......... .......... .......... .......... .......... .......... .......... .......... .......... ..........  
0099B_W-M    .......... .......... .......... .......... .......... .......... .......... .......... .......... .......... .......... .......... .......... ..........  
00912B_W-M   .......... .......... .......... .......... .......... .......... .......... .......... .......... .......... .......... .......... .......... ..........  
0092B_W-M    .......... .......... .......... .......... .......... .......... .......... .......... .......... .......... .......... .......... .......... ..........  
00911B_W-M   .......... .......... .......... .......... .......... .......... .......... .......... .......... .......... .......... .......... .......... ..........  
00914B_W-M   .......... .......... .......... .......... .......... .......... .......... .......... .......... .......... .......... .......... .......... ..........  
0101B_W-M    .......... .......... .......... .......... .......... .......... .......... .......... .......... .......... .......... .......... .......... ..........  
0095B_W-M    .......... .......... .......... .......... .......... .......... .......... .......... .......... .......... .......... .......... .......... ..........  
00910B_W-M   .......... .......... .......... .......... .......... .......... .......... .......... .......... .......... .......... .......... .......... ..........  
0094B_W-M    .......... .........A .......... .......... .......... .......... .......... .......... .......... .......... .......... .......... .......... ..........  
0093B_W-M    .......... .........A .......... .......... .......... .......... .......... .......... .......... .......... .......... .......... .......... ..........  
0096B_W-M    .......... .........A .......... .......... .......... .......... .......... .......... .......... .......... .......... .......... .......... ..........  
0098B_W-M    .......... .........A .......... .......... .......... .......... .......... .......... .......... .......... .......... .......... .......... ..........  
00913B_W-M   .......... .........A .......... .......... .......... .......... .......... .......... .......... .......... .......... .......... .......... ..........  
011TL_L      .......... .........A .......... .......... .A........ .......... .......... .......... .......... .......... .......... .......... .......... ..........  
0083Z_P      .......... .........A .......... .......... .A........ .......... .......... .......... .......... .......... .......... .......... .......... ..........  
019WM_P      .......... .........A .......... .......... .A........ .......... .......... .......... .......... .......... .......... .......... .......... ..........  
0081Z_P      .......... .........A .......... .......... .A........ .......... .......... .......... .......... .......... .......... .......... .......... ..........  
010W_W-M     .......... .........A .......... .......... .A........ .......... .......... .......... .......... .......... .......... .......... .......... ..........  
0409W_W-M    .......... .........A .......... .......... .A........ .......... .......... .......... .......... .......... .......... .......... .......... ..........  
04010W_W-M   .......... .........A .......... .......... .A........ .......... .......... .......... .......... .......... .......... .......... .......... ..........  
0402W_W-M    .......... .........A .......... .......... .A........ .......... .......... .......... .......... .......... .......... .......... .......... ..........  
0405W_W-M    .......... .........A .......... .......... .A........ .......... .......... .......... .......... .......... .......... .......... .......... ..........  
0403W_W-M    .......... .........A .......... .......... .A........ .......... .......... .......... .......... .......... .......... .......... .......... ..........  
0408W_W-M    .......... .........A .......... .......... .A........ .......... .......... .......... .......... .......... .......... .......... .......... ..........  
0407W_W-M    .......... .........A .......... .......... .A........ .......... .......... .......... .......... .......... .......... .......... .......... ..........  
0139O_L_S    .......... .........A .......... .......... .A........ .......... .......... .......... .......... .......... .......... .......... .......... ..........  
0136O_L_S    .......... .........A .......... .......... .A........ .......... .......... .......... .......... .......... .......... .......... .......... ..........  
0138O_L_S    .......... .........A .......... .......... .A........ .......... .......... .......... .......... .......... .......... .......... .......... ..........  
01310O_L_S   .......... .........A .......... .......... .A........ .......... .......... .......... .......... .......... .......... .......... .......... ..........  
0137O_L_S    .......... .........A .......... .......... .A........ .......... .......... .......... .......... .......... .......... .......... .......... ..........  
0133O_L_S    .......... .........A .......... .......... .A........ .......... .......... .......... .......... .......... .......... .......... .......... ..........  
0134O_L_S    .......... .........A .......... .......... .A........ .......... .......... .......... .......... .......... .......... .......... .......... ..........  
0132O_L_S    .......... .........A .......... .......... .A........ .......... .......... .......... .......... .......... .......... .......... .......... ..........  
0135O_L_S    .......... .........A ......G... .......... .A........ .......... .......... .......... .......... .......... .......... .......... .......... ..........  
BLV_FLK      .......... .........A .......... .......... .......... .......... .......... .......... .......... .......... .......... .......... .......... ..........  
11W_W-M      ........GA .......... .......... .......... .......... .......... .......... .......... .......... .......... .......... .......... .......... ..........  
0184S_P      ........GA .......... .......... .......... .......... .......... .......... .......... .......... .......... .......... .......... .......... ..........  
03510M_P     ........GA .......... .......... .......... .......... .......... .......... .......... .......... .......... .......... .......... .......... ..........  
0355M_P      ........GA .......... .......... .......... .......... .......... .......... .......... .......... .......... .......... .......... .......... ..........  
03513M_P     ........GA .......... .......... .......... .......... .......... .......... .......... .......... .......... .......... .......... .......... ..........  
0183S_P      ........GA .......... .......... .......... .......... .......... .......... .......... .......... .......... .......... .......... .......... ..........  
0356M_P      ........GA .......... .......... .......... .......... .......... .......... .......... .......... .......... .......... .......... .......... ..........  
03511M_P     ........GA .......... .......... .......... .......... .......... .......... .......... .......... .......... .......... .......... .......... ..........  
0357M_P      ........GA .......... .......... .......... .......... .......... .......... .......... .......... .......... .......... .......... .......... ..........  
10Sz_W-M     ........GA .......... .......... .......... .......... .......... .......... .......... .......... .......... .......... .......... .......... ..........  
019W_W-M     ........GA ......T... .......... .......... .......... .......... .......... .......... .......... .......... .......C.. .......... .......... ..........  
019L_P       ........GA .......... .......... .......... .......... .......... .......... ...G...... .......... .......... .......... .......... .......... ..........  


                                          TATA Box-binding protein site
                    TRE3    CRE3   Ebox3                                             U3 R
             GRE    ----------------------TATA Box  PAS                    CAP site      +1 
             ----------------             -------   ------                 -------------------  
pBLV344      ACACCCCGAG CTGCTGACCT CACCTGCTGA TAAAACAATA AAATGCCGGC CCTGTCGAGT TAGCGGCACC AGAAGCGTTC TCCTCCTGAG ACCCTCGTGC TCAGCTCTCG GTCCTGAGCT CTCTTGCTCC CGAGACCTTC  
0258G_W-M    ......T... .......... .......... ....TT.... .......... .......... .......... .......... .......... .......... .......... .......... .......... ..........  
0253G_W-M    ......T... .......... .......... ....TT.... .......... ..C....... .......... .......... .......... .......... .......... .......... .......... ..........  
0257G_W-M    ......T... .......... .......... ....TT.... .......... .......... .......... .......... .......... .......... .......... .......... .......... ..........  
0252G_W-M    ......T... .......... .......... ....TT.... .......... .......... .......... .......... .......... .......... .......... .......... .......... ..........  
042AK_K-P    ......T... .......... .G........ ....TT.... .......... .......... .......G.. .......... .......... .......... .......... .......... .......... ..........  
0242K_W-M    ......T... .......... .......... ....TT.... .......... .......... .......... .......... ..T....... .......... .......... .......... .......... ..........  
0256G_W-M    ......T... .......... .......... ....TT.... .......... .......... .......... .......... .......... .......... .......... .......... .......... ..........  
0251G_W-M    ......T... .......... .......... ....TT.... .......... .......... .......... .......... .......... .......... .......... .......... .......... ..........  
0255G_W-M    ......T... .......... .......... ....TT.... .......... .......... .......... .......... .......... .......... .......... .......... .......... ..........  
0741M_S      ......T... .......... .......... ....TT.... .......... .......... -......... .......... .......... .......... .......... .......... .......... ..........  
0133Z_S      ......T... .......... .......... ....TT.... .......... .......... -......... .......... .......... .......... .......... .......... .......... ..........  
0131Z_S      ......T... .......... .......... ....TT.... .......... .......... -......... .......... .......... .......... .......... .......... .......... ..........  
0742M_S      ......T... .......... .......... ....TT.... .......... .......... -......... .......... .......... .......... .......... .......... .......... ..........  
026Z_S       ......T... .......... .......... ....TT.... .......... .......... -......... .......... .......... .......... .......... .......... .......... ..........  
020B_S       ......T... .......... .......... ....TT.... .......... .......... -......... .......... .......... .......... .......... .......... .......... ..........  
0132Z_S      ......T... .......... .......... ....TT.... .......... .......... -......... .......... .......... .......... .......... .......... .......... ..........  
053K_S       ......T... .......... .......... ....TT.... .......... .......... -......... .......... .......... .......... .......... .......... .......... ..........  
0371B_W-M    ......T... .......... .......... ....TT.... .......... .........C .......... .......... .......... .......... .......... .......... .......... ..........  
0374B_W-M    ......T... .......... .......... ....TT.... .......... .........C .......... .......... .......... .......... .......... .......... .......... ..........  
0378B_W-M    ......T... .......... .......... ....TT.... .......... .........C .......... .......... .......... .......... .......... .......... .......... ..........  
3208M_W-M    ......T... .......... .......... ....TT.... .......... .......... .......... .......... .......... .......... .......... .......... .......... ..........  
3175aM_W-M   ......T... .......... .......... ....TT.... .......... .......... .......... .......... .......... .......... .......... .......... .......... ..........  
3205M_W-M    ......T... .......... .......... ....TT.... .......... .......... .......... .......... .......... .......... .......... .......... .......... ..........  
3176aM_W-M   ......T... .......... .......... ....TT.... .......... .......... .......... .......... .......... .......... .......... .......... .......... ..........  
012OM_M      ......T... .......... .......... ....TT.... .......... .......... .......... .......... .......... .......... .......... .......... .......... ..........  
047P_Lodz    ......T... .......... .......... ....TT.... .......... .......... .......... .......... .......... .......... .......... .......... .......... ..........  
0222GD_K-P   ......T... .......... .......... ....TT.... .......... .......... .......... .......... .......... .......... .......... .......... .......... ..........  
0221AGD_K-P  ......T... .......... .......... ....TT.... .......... .......... .......... .......... .......... .......... .......... .......... .......... ..........  
0221GD_K-P   ......T... .......... .......... ....TT.... .......... .......... .......... .......... .......... .......... .......... .......... .......... ..........  
014W_M       ......T... .......... .......... ....TT.... .......... .......... .......... .......... .......... .......... .......... .......... .......... ..........  
014NN_K-P    .......... .......... .......... .......... .......... .......... .......... .......... .......... .......... .......... .......... .C........ ..........  
0166BP_P     .......... ........A. .......... .......... .......... .......... .......... .......... .......... .......... .......... .......... .......... ..........  
01610BP_P    .......... ........A. .......... .......... .......... .......... .......... .......... .......... .......... .......... .......... .......... ..........  
0168BP_P     .......... .......... .......... .......... .......... .......... .......... .......... .......... .......... .......... .......... .......... ..........  
0169BP_P     .......... .......... .......... .......... .......... .......... .......... .......... .......... .......... .......... .......... .......... ..........  
022WM_M      .......... .......... .......... .......... .......... .......... .......... .......... .......... .......... .......... .......... .......... ..........  
0167BP_P     .......... .......... .......... .......... .......... .......... .......... .......... .......... .......... .......... .......... .......... ..........  
4W_W-M       .......... .......... .......... .......... .......... .......... .......... .......... .......... .......... .......... .......... .......... ..........  
0071B_W-M    .......... .......... .......... .......... .......... .......... .......... .......... .......... .......... .......... .......... .......... ..........  
015W_W-M     .......... .......... .......... .......... .......... .......... .......... .......... .......... .......... .......... .......... .......... ..........  
009B_W-M     .......... .......... .......... .......... .......... .......... .......... .......... .......... .......... .......... .......... .......... ..........  
0362B_W-M    .......... .......... .......... .......... .......... .......... .......G.. .......... .......... .......... .......... .......... .......... ..........  
035S_P       .......... .......... .......... .......... .......... .......... .......... .......... .......... .......... .......... .......... .......... ..........  
006B_W-M     .......... .......... .......... .......... .......... .......... .......... .......... .......... .......... .......... .......... .......... ..........  
0244K_W-M    .......... .......... .......... ....T..... .......... .......... .......... .......... .......... .......... .......... .......... .......... ..........  
015P_G_P     .......... .......... .......... ....T..... .......... .......... .......... .......... .......... .......... .......... .......... .......... ..........  
031W_W-M     .......... .......... .......... ....T..... .......... .......... .......... .......... .......... .......... .......... .......... .......... ..........  
017B_W-M     .......... .......... .......... ....T..... .......... .......... .......... .......... .......... .......... .......... .......... .......... ..........  
038W_W-M     .......... .......... .......... ....T..... .......... .......... .......... .....T.... .......... .......... .......... .......... .......... ..........  
00111P_Lodz  .......... .......... ........T. ....T..... .......... .......... .......... .......... .......... ....C..... .......... .......... .......... ...A......  
0018P_Lodz   .......... .......... ........T. ....T..... .......... .......... .......... .......... .......... .......... .......... .......... .......... ...A......  
0057P_Lodz   .......... .......... ........T. ....T..... .......... .......... .......... .......... .......... .......... .......... .......... .......... ...A......  
011L_Lodz    .......... .......... ........T. ....T..... .......... .......... .......... .......... .......... .......... .......... .......... .......... ...A......  
0378B17_W-M  .......... .......... ........T. ....T..... .......... .......... .......... .......... .......... .......... .......... .......... .......... ...A......  
001B_W-M     .......... .......... .......... ....T..... .......... .......... .......... .......... .......... .......... .......... .......... .......... ..........  
002B_W-M     .......... .......... .......... ....T..... .......... .......... .......... .......... .......... .......... .......... .......... .......... ..........  
0072B_W-M    .......... .......... .......... ....T..... .......... .......... .......... .......... .......... .......... .......... .......... .......... ..........  
0102B_W-M    .......... .......... .......... ....T..... .......... .......... .......... .......... .......... .......... .......... .......... .......... ..........  
030O_W-M     .......... .......... .......... ....T..... .......... .......... .......... .......... .......... .......... .......... .......... .......... ..........  
0097B_W-M    .......... .......... .......... ....T..... .......... .......... .......... .......... .......... .......... .......... .......... .......... ..........  
0099B_W-M    .......... .......... .......... ....T..... .......... .......... .......... .......... .......... .......... .......... .......... .......... ..........  
00912B_W-M   .......... .......... .......... ....T..... .......... .......... .......... .......... .......... .......... .......... .......... .......... ..........  
0092B_W-M    .......... .......... .......... ....T..... .......... .......... .......... .......... .......... .......... .......... .......... .......... ..........  
00911B_W-M   .......... .......... .......... ....T..... .......... .......... .......... .......... .......... .......... .......... .......... .......... ..........  
00914B_W-M   .......... .......... .......... ....T..... .......... .......... .......... .......... .......... .......... .......... .......... .......... ..........  
0101B_W-M    .......... .......... .......... ....T..... .......... .......... .......... .......... .......... .......... .......... .......... .......... ..........  
0095B_W-M    .......... .......... .......... ....T..... .......... .......... .......... .......... .......... .......... .......... .......... .......... ..........  
00910B_W-M   .......... .......... .......... ....T..... .......... .......... .......... .......... .......... .......... .......... .......... .......... ..........  
0094B_W-M    .......... .......... .......... ....T..... .......... .......... .......... .......... .......... .......... .......... .......... .......... ..........  
0093B_W-M    .......... .......... .......... ....T..... .......... .......... .......... .......... .......... .......... .......... .......... .......... ..........  
0096B_W-M    .......... .......... .......... ....T..... .......... .......... .......... .......... .......... .......... .......... .......... .......... ..........  
0098B_W-M    .......... .......... .......... ....T..... .......... .......... .......... .......... .......... .......... .......... .......... .......... ..........  
00913B_W-M   .......... .......... .......... ....T..... .......... .......... .......... .......... .......... .......... .......... .......... .......... ..........  
011TL_L      .......... .......... .......... ....TT.... .......... .......... .......... .......... .T........ .......... .......... .......... .......... ..........  
0083Z_P      .......... .......... .......... ....TT.... .......... .......... .......... .......... .T........ .......... .......... .......... .......... ..........  
019WM_P      .......... .......... .......... ....TT.... .......... .......... .......... .......... .T........ .......... .......... .......... .......... ..........  
0081Z_P      .......... .......... .......... ....TT.... .......... .......... .......... .......... .T........ .......... .......... .......... .......... ..........  
010W_W-M     .......... .......... .......... ....TT.... .......... .......... .......... .......... .T........ .......... .......... .......... .......... ..........  
0409W_W-M    .......... .......... .......... ....TT.... .......... .......... .......... .......... .T........ .......... .......... .......... .......... ..........  
04010W_W-M   .......... .......... .......... ....TT.... .......... .......... .......... .......... .T........ .......... .......... .......... .......... ..........  
0402W_W-M    .......... .......... .......... ....TT.... .......... .......... .......... .......... .T........ .......... .......... .......... .......... ..........  
0405W_W-M    .......... .......... .......... ....TT.... .......... .......... .......... .......... .T........ .......... .......... .......... .......... ..........  
0403W_W-M    .......... .......... .......... ....TT.... .......... .......... .......... .......... .T........ .......... .......... .......... .......... ..........  
0408W_W-M    .......... .......... .......... ....TT.... .......... .......... .......... .......... .T........ .......... .......... .......... .......... ..........  
0407W_W-M    .......... .......... .......... ....TT.... .......... .......... .......... .......... .T........ .......... .......... .......... .......... ..........  
0139O_L_S    .......... .......... .......... ....TT.... .......... .......... .......... .......... .T........ .......... .......... .......... .......... ..........  
0136O_L_S    .......... .......... .......... ....TT.... .......... .......... .......... .......... .T........ .......... .......... .......... .......... ..........  
0138O_L_S    .......... .......... .......... ....TT.... .......... .......... .......... .......... .T........ .......... .......... .......... .......... ..........  
01310O_L_S   .......... .......... .......... ....TT.... .......... .......... .......... .......... .T........ .......... .......... .......... .......... ..........  
0137O_L_S    .......... .......... .......... ....TT.... .......... .......... .......... .......... .T........ .......... .......... .......... .......... ..........  
0133O_L_S    .......... .......... .......... ....TT.... .......... .......... .......... .......... .T........ .......... .......... .......... .......... ..........  
0134O_L_S    .......... .......... .......... ....TT.... .......... .......... .......... .......... .T........ .......... .......... .......... .......... ..........  
0132O_L_S    .......... .......... .......... ....TT.... .......... .......... .......... .......... .T........ .......... .......... .......... .......... ..........  
0135O_L_S    .......... .......... .......... ....TT.... .......... .......... .......... .......... .T........ .......... .......... .......... .......... ..........  
BLV_FLK      ......T... .......... .......... ....TT.... .......... .......... .......... .......... .T........ .......... .......... .......... .......... ..........  
11W_W-M      ......T... .......... .......... A...TT.... .......... .......... .......... .......... .T........ .......... .......... .......... .......... ..........  
0184S_P      ......T... .......... .......... A...TT.... .......... .......... .......... .......... .T........ .......... .......... .......... .......... ..........  
03510M_P     ......T... .......... .......... A...TT.... .......... .......... .......... .......... .T........ .......... .......... .......... .......... ..........  
0355M_P      ......T... .......... .......... A...TT.... .......... .......... .......... .......... .T........ .......... .......... .......... .......... ..........  
03513M_P     ......T... .......... .......... A...TT.... .......... .......... .......... .......... .T........ .......... .......... .......... .......... ..........  
0183S_P      ......T... .......... .......... A...TT.... .......... .......... .......... .......... .T........ .......... .......... .......... .......... ..........  
0356M_P      ......T... .......... .......... A...TT.... .......... .......... .......... .......... .T........ .......... .......... .......... .......... ..........  
03511M_P     ......T... .......... .......... A...TT.... .......... .......... .......... .......... .T........ .......... .......... .......... .......... ..........  
0357M_P      ......T... .......... .......... A...TT.... .......... .......... .......... .......... .T........ .......... .......... .......... .......... ..........  
10Sz_W-M     ......T... .......... .......... A...TT.... .......... .......... .......... .......... .T........ .......... .......... .......... .......... ..........  
019W_W-M     ......T... .......... .......... A...TT.... .......... .......... .......... .......... .T........ .......... .......... .......... .......... ..........  
019L_P       ......T... .......... .......... A...TT.... .......... .......... .......... .......... .T........ .......... .......... .......... ...C...... ..........  


                                                                                                      DAS
                                                                                               Box A            Box B       USF          Box C                                           
                                                                                                ------------------------------------------------------------------- 
pBLV344      TGGTCGGCTA TCCGGCAGCG GTCAGGTAAG GCAAACCACG GTTTGGAGGG TGGTTCTCGG CTGAGACCAC CGCGAGCTCT ATCTCCGGTC CTCTGACCGT CTCCACGTGG ACTCTCTCTC T--TGCCTCC TGACCCCGCG  
0258G_W-M    .......... .......... .......... .......... .......... .......... .......... .......... .......... .......... .......... .......... .CT....... ..........  
0253G_W-M    .......... .......... .......... .......... .......... .......... .......... .......... .......... .......... .......... .......... .CT....... ..........  
0257G_W-M    .......... .......... .......... .......... .......... .......... .......... .......... .......... .......... .......... .......... .CT....... ..........  
0252G_W-M    .......... .......... .......... .......... .......... .......... .......... .......... .......... .......... .......... .......... .CT....... ..........  
042AK_K-P    .......... .......... .......... .......... .......... ..A....... .......... .......... .......... .......... .......... .......... .--....... ..........  
0242K_W-M    .......... .......... .......... .......... .......... .......... .......... .......... .......... .......... .......... .......... .--....... ..........  
0256G_W-M    .......... .......... .......... .......... .......... .......... .......... .......... .......... .......... .......... .......... .CT....... ..........  
0251G_W-M    .......... .......... .......... .......... .......... .......... .......... .......... .......... .......... .......... .......... .CT....... ..........  
0255G_W-M    .......... .......... .......... .......... .......... .......... .......... .......... .......... .......... .......... .......... .CT....... ..........  
0741M_S      .......... .......... .......... .......... .......... .......... .......... .......... .......... .......... .......... .......... .--....... ..........  
0133Z_S      .......... .......... .......... .......... .......... .......... .......... .......... .......... .......... .......... .......... .--....... ..........  
0131Z_S      .......... .......... .......... .......... .......... .......... .......... .......... .......... .......... .......... .......... .--....... ..........  
0742M_S      .......... .......... .......... .......... .......... .......... .......... .......... .......... .......... .......... .......... .--....... ..........  
026Z_S       .......... .......... .......... .......... .......... .......... .......... .......... .......... .......... .......... .......... .--....... ..........  
020B_S       .......... .......... .......... .......... .......... .......... .......... .......... .......... .......... .......... .......... .--....... ..........  
0132Z_S      .......... .......... .......... .......... .......... .......... .......... .......... .......... .......... .......... .......... .--....... ..........  
053K_S       .......... .......... .......... .......... .......... .......... .......... .......... .......... .......... .......... .......... .--....... ..........  
0371B_W-M    .......... .......... .......... .......... .......... .......... .......... .......... .......... .......... .......... .......... .--....... ..........  
0374B_W-M    .......... .......... .......... .......... .......... .......... .......... .......... .......... .......... .......... .......... .--....... ..........  
0378B_W-M    .......... .......... .......... .......... .......... .......... .......... .......... .......... .......... .......... .......... .--....... ..........  
3208M_W-M    .......... .......... .......... .......... .......... .......... .......... .......... .......... .......... .......... .......... .--....... ..........  
3175aM_W-M   .......... .......... .......... .......... .......... .......... .......... .......... .......... .......... .......... .......... .--....... ..........  
3205M_W-M    .......... .......... .......... .......... .......... .......... .......... .......... .......... .......... .......... .......... .--....... ..........  
3176aM_W-M   .......... .......... .......... .......... .......... .......... .......... .......... .......... .......... .......... .......... .--....... ..........  
012OM_M      .......... .......... .......... .......... .......... .......... .......... .......... .......... .......... .......... ..C....... .--....... ..........  
047P_Lodz    .......... .......... .......... .......... .......... .......... .......... .......... .......... .......... .......... ..C....... .--....... ..........  
0222GD_K-P   .......... .......... .......... .......... .......... .......... .......... .A........ .......... .......... .......... .......... .--....... ..........  
0221AGD_K-P  .......... .......... .......... .......... .......... .......... .......... .A........ .......... .......... .......... .......... .--....... ..........  
0221GD_K-P   .......... .......... .......... .......... .......... .......... .......... .A........ .......... .......... .......... .......... .--....... ..........  
014W_M       .......... .......... .......... .......... .......... .......... .......... .......... .......... .......... .......... .......... .--....... ..........  
014NN_K-P    .......... .......... .......... .......... .......... C......... .......... .......... .......... .......... .......... .......... .--....... ..........  
0166BP_P     .......... .......... .......... .......... ...G...... ...C...... .......... .......... .......... .......... .......... .......... .--....... ..........  
01610BP_P    .......... .......... .......... .......... ...G...... ...C...... .......... .......... .......... .......... .......... .......... .--....... ..........  
0168BP_P     .......... .......... .......... .......... ...G...... ...C...... .......... .......... .......... .......... .......... .......... .--....... ..........  
0169BP_P     .......... .......... .......... .......... ...G...... ...C...... .......... .......... .......... .......... .......... .......... .--....... ..........  
022WM_M      .......... .......... .......... .......... ...G...... ...C...... .......... .......... .......... .......... .......... .......... .--....... ..........  
0167BP_P     .......... .......... .......... .......... ...A...... ...C...... .......... .......... .......... .......... .......... .......... .--....... ..........  
4W_W-M       .......... .......... .......... .......... .......... .......... .......... .......... .......... .......... .......... .......... .--....... ..........  
0071B_W-M    .......... .......... .......... .......... .......... .......... .......... .......... .......... .......... .......... .......... .--....... ..........  
015W_W-M     .......... .......... .......... .......... .......... .......... .......... .......... .......... .......... .......... .......... .--....... ..........  
009B_W-M     .......... .......... .......... .......... .......... .......... .......... .......... .......... .......... .......... .......... .--....... ..........  
0362B_W-M    .......... .......... .......... .......... .......... .......... .......... .......... .......... .......... .......... .......... .--....... ..........  
035S_P       .......... .......... .......... .......... .......... .......... .......... .......... .......... .......... .......... .........- ---....... ..........  
006B_W-M     .......... .......... .......... .......... .......... .......... .......... .......... .......... .......... .......... .......... .--....... ..........  
0244K_W-M    .......... .......... .......... .......... .......... .......... .......... .......... .......... .......... .......... .......... .CT....... ..........  
015P_G_P     .......... .......... .......... .......... .......... .......... .......... .......... .......... .......... .......... .......... .--....... ..........  
031W_W-M     .......... .......... .......... .......... .......... .......... .......... .......... .......... .......... .......... .......... .--....... ..........  
017B_W-M     .......... .......... .......... .......... .......... .......... .......... .......... .......... .......... .......... .......... .--....... ..........  
038W_W-M     .......... .......... .......... .......... .......... .......... .......... .......... .......... .......... .......... .......... .--....... ..........  
00111P_Lodz  .......... .......... .......... .......... .......... .......... .......... .......... .......... .......... .......... .......... .--....... ..........  
0018P_Lodz   .......... .......... .......... .......... .......... .......... .......... .......... .......... .......... .......... .......... .--....... ..........  
0057P_Lodz   .......... .......... .......... .......... .......... .......... .......... .......... .......... .......... .......... .......... .--....... ..........  
011L_Lodz    .......... .......... .......... .......... .......... .......... .......... .......... .......... .......... .......... .......... .--....... ..........  
0378B17_W-M  .......... .......... .......... .......... .......... .......... .......... .......... .......... .......... .......... .......... .--....... ..........  
001B_W-M     .......... .......... .......... .......... .......... .......... .......... .......... .......... .......... .......... .......... .CT....... ..........  
002B_W-M     .......... .......... .......... .......... .......... .......... .......... .......... .......... .......... .......... .......... .CT....... ..........  
0072B_W-M    .......... .......... .......... .......... .......... .......... .......... .......... .......... .......... .......... .......... .--....... ..........  
0102B_W-M    .......... .......... .......... .......... .......... .......... .......... .......... .......... .......... .......... .......... .--....... ..........  
030O_W-M     .......... .......... .......... .......... .......... .......... .......... .......... .......... .......... .......... .......... .--....... ..........  
0097B_W-M    .......... .......... .......... .......... .......... .......... .......... .......... .......... .......... .......... .......... C--....... ..........  
0099B_W-M    .......... .......... .......... .......... .......... .......... .......... .......... .......... .......... .......... .......... C--....... ..........  
00912B_W-M   .......... .......... .......... .......... .......... .......... .......... .......... .......... .......... .......... .......... C--....... ..........  
0092B_W-M    .......... .......... .......... .......... .......... .......... .......... .......... .......... .......... .......... .......... C--....... ..........  
00911B_W-M   .......... .......... .......... .......... .......... .......... .......... .......... .......... .......... .......... .......... C--....... ..........  
00914B_W-M   .......... .......... .......... .......... .......... .......... .......... .......... .......... .......... .......... .......... C--....... ..........  
0101B_W-M    .......... .......... .......... .......... .......... .......... .......... .......... .......... .......... .......... .......... C--....... ..........  
0095B_W-M    .......... .......... .......... .......... .......... .......... .......... .......... .......... .......... .......... .......... C--....... ..........  
00910B_W-M   .......... .......... .......... .......... .......... .......... .......... .......... .......... .......... .......... .......... C--....... ..........  
0094B_W-M    .......... .......... .......... .......... .......... .......... .......... .......... .......... .......... .......... .......... C--....... ..........  
0093B_W-M    .......... .......... .......... .......... .......... .......... .......... .......... .......... .......... .......... .......... C--....... ..........  
0096B_W-M    .......... .......... .......... .......... .......... .......... .......... .......... .......... .......... .......... .......... C--....... ..........  
0098B_W-M    .......... .......... .......... .......... .......... .......... .......... .......... .......... .......... .......... .......... C--....... ..........  
00913B_W-M   .......... .......... .......... .......... .......... .......... .......... .......... .......... .......... .......... .......... C--....... ..........  
011TL_L      .......... ..T....... .......... .......... .......... .......... .......... .......... .......... .......... .......... ........CT .--....... ..........  
0083Z_P      .......... .......... .......... .......... .......... .......... .......... .......... .......... .......... .......... ........CT .--....... ..........  
019WM_P      .......... .......... .......... .......... .......... .......... .......... .......... .......... .......... .......... ........CT .--....... ..........  
0081Z_P      .......... .......... .......... .......... .......... .......... .......... .......... .......... .......... .......... ........CT .--....... ..........  
010W_W-M     .......... .......... .......... .......... .......... .......... .......... ......T... ......A... .......... .......... ........CT .--....... ..........  
0409W_W-M    .......... .......... .......... .......... .......... .......... .......... .......... .......... .......... .......... ........CT .--....... ..........  
04010W_W-M   .......... .......... .......... .......... .......... .......... .......... .......... .......... .......... .......... ........CT .--....... ..........  
0402W_W-M    .......... .......... .......... .......... .......... .......... .......... .......... .......... .......... .......... ........CT .--....... ..........  
0405W_W-M    .......... .......... .......... .......... .......... .......... .......... .......... .......... .......... .......... ........CT .--....... ..........  
0403W_W-M    .......... .......... .......... .......... .......... .......... .......... .......... .......... .......... .......... ........CT .--....... ..........  
0408W_W-M    .......... .......... .......... .......... .......... .......... .......... .......... .......... .......... .......... ........CT .--....... ..........  
0407W_W-M    .......... .......... .......... .......... .......... .......... .......... .......... .......... .......... .......... ........CT .--....... ..........  
0139O_L_S    .......... .......... .......... .......... .......... .......... .......... ..T....... .......... .......... .......... ........CT .--....... ..........  
0136O_L_S    .......... .......... .......... .......... .......... .......... .......... ..T....... .......... .......... .......... ........CT .--....... ..........  
0138O_L_S    .......... .......... .......... .......... .......... .......... .......... ..T....... .......... .......... .......... ........CT .--....... ..........  
01310O_L_S   .......... .......... .......... .......... .......... .......... .......... .......... .......... .......... .......... ........CT .--....... ..........  
0137O_L_S    .......... .......... .......... .......... .......... .......... .......... .......... .......... .......... .......... ........CT .--....... ..........  
0133O_L_S    .......... .......... .......... .......... .......... .......... .......... .......... .......... .......... .......... ........CT .--....... ..........  
0134O_L_S    .......... .......... .......... .......... .......... .......... .......... .......... .......... .......... .......... ........CT .--....... ..........  
0132O_L_S    .......... .......... .......... .......... .......... .......... .......... .......... .......... .......... .......... ........CT .--....... ..........  
0135O_L_S    .......... .......... .......... .......... .......... .......... .......... .......... .......... .......... .......... ........CT .--....... ..........  
BLV_FLK      .......... .......... .......... .......... .......... .......... .......... .......... .......... .......... .......... ........CT .--....... ..........  
11W_W-M      .......... .......... .......... .......... .......... .......... .......... .......... G......... .......... .......... .......... .--....... ..........  
0184S_P      .......... .......... .......... .......... .......... .......... .......... .......... G......... .......... .......... .......... .--....... ..........  
03510M_P     .......... .......... .......... .......... .......... .......... .......... .......... G......... .......... .......... .......... .--....... ..........  
0355M_P      .......... .......... .......... .......... .......... .......... .......... .......... G......... .......... .......... .......... .--....... ..........  
03513M_P     .......... .......... .......... .......... .......... .......... .......... .......... G......... .......... .......... .......... .--....... ..........  
0183S_P      .......... .......... .......... .......... .......... .......... .......... .......... G......... .......... .......... .......... .--....... ..........  
0356M_P      .......... .......... .......... .......... .......... .......... .......... .......... G......... .......... .......... .......... .--....... ..........  
03511M_P     .......... .......... .......... .......... .......... .......... .......... .......... G......... .......... .......... .......... .--....... ..........  
0357M_P      .......... .......... .......... .......... .......... .......... .......... .......... G......... .......... .......... .......... .--....... ..........  
10Sz_W-M     .......... .......... .......... .......... .......... .......... .......... .......... G......... .......... .......... ...T...... .--....... ..........  
019W_W-M     .......... .......... .......... .......... .......... .......... .......... .......... G......... .......... .......... .......... .--....... ..........  
019L_P       .......... .......... .......... .......... .......... .......... .......... .......... G......... .......... .......... .......... .--....... ..........  


                                   --                      IRF
             --                          R U5           -----------------                                                          U5
pBLV344      CTCCAAGGGC GTCTGGCTTG CACCCGCGCT TGTTTCCTGT CTTACTTTCT GTTTCTCGCG GCCCGCGCTC TCTCCTTCGG CGCCCTCTAG CGGCCAGGAG AGACCGGCAA ACA 
0258G_W-M    .......... .......... .......... .......... .......... .......... .......... .......... .......... .......... .......... ... 
0253G_W-M    .......... .......... .......... .......... .......... .......... .......... .......... .......... .......... .......... ... 
0257G_W-M    .......... .......... .......... .......... .......... .......... .......... .......... .......... .......... .......... ... 
0252G_W-M    .......... .......... .......... .......... .......... .......... .......... .......... .......... .......... .......... ... 
042AK_K-P    .......... .......... .......A.. .......... .......... .......... .......... .......... .......... .......... .......... ... 
0242K_W-M    .......... .......... .......... .......... .......... .......... .......... .......... .......... .......... .......... ... 
0256G_W-M    .......... .......... .......... .......... .......... .......... .......... .......... .......... .......... .......... ... 
0251G_W-M    .......... .......... .......... .......... .......... .......... .......... .......... .......... .......... .......... ... 
0255G_W-M    .......... .......... .......... .......... .......... .......... .......... .......... .......... .......... .......... ... 
0741M_S      .......... .......... .......... .......... .......... .......... .......... .......... .......... .......... .......... ... 
0133Z_S      .......... .......... .......... .......... .......... .......... .......... .......... .......... .......... .......... ... 
0131Z_S      .......... .......... .......... .......... .......... .......... .......... .......... .......... .......... .......... ... 
0742M_S      .......... .......... .......... .......... .......... .......... .......... .......... .......... .......... .......... ... 
026Z_S       .......... .......... .......... .......... .......... .......... .......... .......... .......... .......... .......... ... 
020B_S       .......... .......... .......... .......... .......... .......... .......... .......... .......... .......... .......... ... 
0132Z_S      .......... .......... .......... .......... .......... .......... .......... .......... .......... .......... .......... ... 
053K_S       .......... .......... .......... .......... .......... .......... .......... .......... .......... ........G. .......... ... 
0371B_W-M    .......... .......... .......... .......... .......... .......... .......... .......... .......... T....T.... .......... ... 
0374B_W-M    .......... .......... .......... .......... .......... .......... .......... .......... .......... T....T.... .......... ... 
0378B_W-M    .......... .......... .......... .......... .......... .......... .......... .......... .......... T......... .......... ... 
3208M_W-M    .......... .......... .......... .......... .......... .......... .....T..C. .......... .......... .....T.... .......... ... 
3175aM_W-M   .......... .......... .......... .......... .......... .......... .....T..C. .......... .......... .....T.... .......... ... 
3205M_W-M    .......... .......... .......... .......... .......... .......... .....T..C. .......... .......... .....T.... .......... ... 
3176aM_W-M   .......... .......... .......... .......... .......... .......... .....T..C. .......... .......... .....T.... .......... ... 
012OM_M      .......... .......... .......... .......... .......... .......... .......... .......... .......... .....T.... .......... ... 
047P_Lodz    .......... .......... .......... .......... .......... .......... .......... .......... .......... .....T.... .......... ... 
0222GD_K-P   .......... .......... .......... .......... .......... .......... .......... .......... .......... .....T.... .......... ... 
0221AGD_K-P  .......... .......... .......... .......... .......... .......... .......... .......... .......... .......... .......... ... 
0221GD_K-P   .......... .......... .......... .......... .......... .......... .......... .......... .......... .....T.... .......... ... 
014W_M       .......... .......... .......... .......... .......... .......... .......... ..C....... .......... .......... .......... ... 
014NN_K-P    .......... .......... ......A... .......... .......... .......... .......... .......... .........A .......... .......... ... 
0166BP_P     .......... .......... ...T...... .......... .......... .......... .......... .......... .......... .......... .......... ... 
01610BP_P    .......... .......... ...T...... .......... .......... .......... .......... .......... .......... .......... .......... ... 
0168BP_P     .......... .......... ...T...... .......... .......... .......... .......... C......... .......... .......... .......... ... 
0169BP_P     .......... .......... ...T...... .......... .......... .......... .......... .......... .......... .......... .......... ... 
022WM_M      .......... .......... ...T...... .......... .......... .......... .......... .......... .......... .......... .......... ... 
0167BP_P     .......... .......... ...T...... .......... .......... .......... .......... .......... .......... .......... .......... ... 
4W_W-M       .......... .......... .......... .......... .......... .......... .......... .......... .......... .......... .......... ... 
0071B_W-M    .......... .......... .......... .......... .......... .......... .......... .......... .......... .......... .......... ... 
015W_W-M     .......... .......... .......... .......... .......... .......... .......... .......... .......... .......... .......... ... 
009B_W-M     .......... .......... .......... .......... .......... .......... .......... .......... .......... .......... .......... ... 
0362B_W-M    .......... .......... .......... .......... .......... .......... .......... .......... .......... .......... .........G ... 
035S_P       .......... .......... .......... .......... .......... .......... .......... .......... .......... .......... .......... ... 
006B_W-M     .......... .......... .......... .........A .......... .......... .......... .......... T......... .......... .......A.. ... 
0244K_W-M    .......... .......... .......... .......... .......... .......... .......... .......... .......... .......... ......CA.C ... 
015P_G_P     .......... .......... .......... .......C.. .......... .......... .......... .......... .......... .......... .......... ... 
031W_W-M     .......... .......... .......... .......... .......... .......... .......... .....C.... .......... ....-..... .......... ... 
017B_W-M     .......... .......... .......... .......... .......... .......... .......... .......... .......... .......... .......... ... 
038W_W-M     .......... .......... .......... .......... .......... .......... .......... .......... .......... .......... .......... ... 
00111P_Lodz  .C........ .......... .......... .......... .......... .......... .......... .......... .......... .......... .......... ... 
0018P_Lodz   .......... .......... .......... .......... .......... .......... .......... .......... .......... .......... .......... ... 
0057P_Lodz   .......... .......... .......... .......... .......... .......... .......... .......... .......... .......... .......... ... 
011L_Lodz    .......... .......... .......... .......... .......... .......... .......... .......... .......... .......... .......... ... 
0378B17_W-M  .......... .......... .......... .......... .......... .......... .......... .......... .......... .......... .......... ... 
001B_W-M     .......... .......... .......... .......... .......... .......... .......... .......... .......... .......... .......... ... 
002B_W-M     .......... .......... .......... .......... .......... .......... .......... .......... .......... .......... .......... ... 
0072B_W-M    .......... .......... .......... .......... .......... .......... .......... .......... .......... .......... .......... ... 
0102B_W-M    .......... .......... .......... .......... .......... .......... .......... .......... .......... .......... .......... ... 
030O_W-M     .......... .......... .......... .......... .......... ....T..... .......... .......... .......... .......... .......... ... 
0097B_W-M    .......... .......... .......... .......... .......... .......... .......... .......... .......... .......... .......... ... 
0099B_W-M    .......... .......... .......... .......... .......... .......... .......... .......... .......... .......... .......... ... 
00912B_W-M   .......... .......... .......... .......... .......... .......... .......... .......... .......... .......... .......... ... 
0092B_W-M    .......... .......... .......... .......... .......... .......... .......... .......... .......... .......... .......... ... 
00911B_W-M   .......... .......... .......... .......... .......... .......... .......... .......... .......... .......... .......... ... 
00914B_W-M   .......... .......... .......... .......... .......... .......... .......... .......... .......... .......... .......... ... 
0101B_W-M    .......... .......... .......... .......... .......... .......... .......... .......... .......... .......... .......... ... 
0095B_W-M    .......... .......... .......... .......... .......... .......... .......... .......... .......... .......... .......... ... 
00910B_W-M   .......... .......... .......... .......... .......... .......... .......... .......... .......... .......... .......... ... 
0094B_W-M    .......... .......... .......... .......... .......... .......... .......... .......... .......... .......... .......... ... 
0093B_W-M    .......... .......... .......... .......... .......... .......... .......... .......... .......... .......... .......... ... 
0096B_W-M    .......... .......... .......... .......... .......... .......... .......... .......... .......... .......... .......... ... 
0098B_W-M    .......... .......... .......... .......... .......... .......... .......... .......... .......... .......... .......... ... 
00913B_W-M   .......... .......... .......... .......... .......... .......... .......... .......... .......... .......... .......... ... 
011TL_L      .......... .......... ........T. C......... .......... .......... .......... .......... .......... .......... .......... ... 
0083Z_P      .......... .......... ........T. C......... .......... .......... .......... .......... .......... .......... .......... ... 
019WM_P      .......... .......... ........T. C......... .......... .......... .......... .......... .......... .......... .......... ... 
0081Z_P      .......... .......... ........T. C......... .......... .......... .......... .......... .......... .......... .......... ... 
010W_W-M     .......... .......... ........T. C......... .......... .......... .......... .....C.... .......... .......... .......... ... 
0409W_W-M    .......... .......... ........T. C......... .......... .......... .......... .....C.... .......... .......... .......... ... 
04010W_W-M   .......... .......... ........T. C......... .......... .......... .......... .....C.... .......... .......... .......... ... 
0402W_W-M    .......... .......... ........T. C......... .......... .......... .......... .....C.... .......... .......... .......... ... 
0405W_W-M    .......... .......... ........T. C......... .......... .......... .......... .....C.... .......... .......... .......... ... 
0403W_W-M    .......... .......... ........T. C......... .......... .......... .......... .....C.... .......... .......... .......... ... 
0408W_W-M    .......... .......... ........T. C......... .......... .......... .......... .....C.... .......... .......... .......... ... 
0407W_W-M    .......... .......... ........T. C......... .......... .......... .......... .....C.... .......... .......... .......... ... 
0139O_L_S    .......... .......... ........T. C......... .......... .......... .......... ......C... .......... .......... .......... CA. 
0136O_L_S    .......... .......... ........T. C......... .......... .......... .......... ......C... .......... .......... .......... CA. 
0138O_L_S    .......... .......... ........T. C......... .......... .......... .......... ......C... .......... .......... .......... CA. 
01310O_L_S   .......... .......... ........T. C......... ......C... .......... .......... ......C... .......... .......... .......... CA. 
0137O_L_S    .......... .......... ........T. C......... ......C... .......... .......... ......C... .......... .......... .......... CA. 
0133O_L_S    .......... .......... ........T. C......... ......C... .......... .......... ......C... .......... .......... .......... CA. 
0134O_L_S    .......... .......... ........T. C......... .......... .......... .......... ......C... .......... .......... .......... CA. 
0132O_L_S    .......... .......... ........T. C......... .......... .......... .......... ......C... .......... .......... .......... CA. 
0135O_L_S    .......... .......... ........T. C......... .......... .......... .......... ......C... .......... .......... .......... CA. 
BLV_FLK      .......... .......... ........T. .......... .......... .......... .......... .......... .......... .......... .......... ... 
11W_W-M      .......... .......... .......... .......... .......... .........A .......... .....C.... .......C.. .......... .......... ... 
0184S_P      .......... .......... .......... .......... .......... .........A .......... .....C.... .......C.. .......... .......... ... 
03510M_P     .......... .......... .......... .......... .......... .........A .......... .....C.... .......C.. .......... .......... ... 
0355M_P      .......... .......... .......... .......... .......... .........A .......... .....C.... .......C.. .......... .......... ... 
03513M_P     .......... .......... .......... .......... .......... .........A .......... .....C.... .......C.. .......... .......... ... 
0183S_P      .......... .......... .......... .......... .......... .........A .......... .....C.... .......C.. .......... .......... ... 
0356M_P      .......... .......... .......... .......... .......... .........A .......... .....C.... .......C.. .......... .......... ... 
03511M_P     .......... .......... .......... .......... .......... .........A .......... .....C.... .......C.. .......... .......... ... 
0357M_P      .......... .......... .......... .......... .......... .........A .......... .....C.... .......C.. .......... .......... ... 
10Sz_W-M     .......... .......... .......... .......... .......... .......... .......... .....C.... .......C.. .......... .......... ... 
019W_W-M     .......... .......... .......... .......... .......... .......... .......... .....C.... .......C.. .......... .......... ... 
019L_P       .......... .......... .......... .......... .......... .........A .......... .....C...A TA......G. T......... .......... ... 

Figure S1: Alignment of LTR region nucleotide sequences of 106 Polish BLV strains. Divergence from the reference strain 344 sequence are indicated. Distribution of corresponding regulatory elements along the LTR are indicated in the header. Horizontal dashed lines above the nucleotide sequence alignment indicate the TxRE, Ebox, TATA box, êB, GRE, PU.1/Spi-B, CAT, CAP site, DAS, and IRF. Double line below the strain 344 sequence indicate TATA Box-binding protein site; a single line below the strain 344 sequence indicate the êB-like site; boxes from A to C within DAS and USF. The most frequent changes located in the regulatory elements in the U3 and R region were marked in blue. The changes that created a putative MAZ site were marked in yellow.

Figure S2: Phylogenetic analysis of LTR sequences. Phylogenetic relationship of a 531-bp region of the 106 LTR nucleotide sequences, BLV-FLK sequence and strain 344 (pBLV344) (n = 108), as inferred by Bayesian analysis. Numbers at nodes indicate posterior probabilities of sampling the node among 10,000 trees. Subtype terminology is based on that of Pluta et al. [36]. LTR sequence variants selected for further study are indicated at the right by red squares.

                                                   Zn finger domain
                                               ----------------------------                           
BLV_strain_344  MASVVGWGPH SLHACPALVL SNDVTIDAWC PLCGPHERLQ FERIDTTLTC ETHRITWTAD GRPFGLNGTL FPRLHVSETR 80 
1) 0137O_L_S       .......... .......... .......... .......... .......... .......... ........A. ..........  
2) 011TL_L         .......... .......... .......... .......... .......... .......... ........A. ..........  
3) 0138O_L_S       .......... .......... .......... .......... .......... .......... ........A. ..........  
4) 0135O_L_S       .......... .......... .......... .......... .......... .......... ........A. ..........  
5) 0403W_W-M       .......... .......... .......... .......... .......... .......... ........A. ..........  
6) 10Sz_W-M        .......... .......... .......... .......... .......... .......... ........A. .........H  
7) 11W_W-M         .......... .......... .......... .......... .......... .......... ........A. ..........  
8) 0357M_P         .......... .......... .......... .......... .......... .......... ........A. ..........  
9) 019L_P          .......... .......... .......... .......... .......... .......... ........A. ..........  
10)0355M_P         .......... .......... .......... .......... .......... .......... ........A. ..........  
11)019W_W-M        .......... .......... .......... .......... .......... .......... ........A. ..........  
12)0378B_W-M       .......... .......... .......... .......... .......... .......... .......... ..........  
13)012OM_M         .......... .......... .......... .......... .......... .......... .......... ..........  
14)0221AGD_K-P     .......... .......... .......... .......... .K........ .......... .......... ..........  
15)0222GD_K_P      .......... .......... .......... .......... .K........ .......... .......... ..........  
16)009B_W-M        .......... .......... .......... .......... .......... .......... .......... ..........  
17)4W_W-M          .......... .......... .......... .......... .......... .......... .......... ..........  
18)015W_W-M        .......... .......... .......... .......... .......... .......... .......... ..........  
19)0741M_S         .......... .......... .......... .......... .......... .......... ...Y...... ..........  
20)0742M_S         .......... .......... .......... .......... .......... .......... ...Y...... ..........  
21)0131Z_S         .......... .......... .......... .......... .......... .......... ...Y...... ..........  
22)0133Z_S         .......... .......... .......... .......... .......... .......... ...Y...... ..........  
23)026Z_S          .......... .......... .......... .......... .......... .......... ...Y...... ..........  
24)020B_S          .......... .......... .......... .......... .......... .......... ...Y...... ..........  
25)042AK_K-P       .......... .......... .......... .......... .......... .......... ...Y....M. ..........  
26)0257G_W-M       .......... .......... .......... .......... .......... .......... ........M. ..........  
27)0253G_W-M       .......... .......... .......... .......... .......... .......... ........M. ..........  
28)0258G_W-M       .......... .......... .......... .......... .......... .......... ........M. ..........  
29)014W_M          .......... .......... .......... .......... .......... .......... ........M. ..........  
30)017B_W-M        .......... .......... .......... .......... .......... .......... .......... ..........  
31)002B_W-M        .......... .......... .......... .......... .......... .......... .......... .........H  
32)001B_W-M        .......... .......... .......... .......... .......... .......... .......... .........H  
33)035S_P          .......... .......... .......... .......... .......... .......... ........M. ..........  
34)0072B_W-M       .......... .......... .......... .......... .......... .......... ........M. ..........  
35)00913B_W-M      .......... .......... .......... .......... .......... .......... ........M. ..........  
36)0094B_W-M       .......... .......... .......... .......... .......... .......... ........M. ..........  
37)0018P_Lodz      .......... .......... .......... .......... .......... .......... ........M. ..........  
38)011L_Lodz       .......... .......... .......... .......... .......... .......... ........M. ..........  
39)00111P_Lodz     .......... .......... .......... .......... .......... .......... ........M. ..........  
40)01610BP_P       .......... .......... .......... .......... .......... .......... ........M. ..........  
41)0169BP_P        .......... .......... .......... .......... .......... .......... ........M. ..........  
42)0168BP_P        .......... .......... .......... .......... .......... .......... ........M. ..........  
43)031W_W-M        .......... .......... .......... .......... .......... .......... ........M. ..........  
44)BLV_FLK         .......... .......... .......... .......... .......... .....N.... ...C...... ..........  


                                                                                              CD8+ CTL
                                                  T-cell epitopes                             ---------         
                                           +      ------------------------------------------       ----
BLV_strain_344  PQGPRRLWIN CPLPAVRAQP GPVSLSPFEQ SPFQPYQCQL PSASSDGCPI IGHGLLPWNS LVTHPVLGKV LTLNQMANFS 160 
0137O_L_S       .......... .......... .........R .......... .......... .........N .......... .I........  
011TL_L         .......... .......... .........R .......... .......... .........N .......... .I........  
0138O_L_S       .......... .......... .........R .......... .......... .........N .......... .I........  
0135O_L_S       .......... .......... .........R .......... .......... .........N .......... .I........  
0403W_W-M       .......... .......... .........R .......... ...F...... .........N .......... .I........  
10Sz_W-M        .......... ....T..... .......... .......... .......... .........N .......... .I........  
11W_W-M         .......... ....T..... .......... .......... .......... .........N .......... .I........  
0357M_P         .......... ....T..... .......... .......... .......... .........N .......... .I........  
019L_P          .......... ....T..... .......... .......... .......... .........N .......... .I........  
0355M_P         .......... ....T..... .......... .......... .......... .........N .......... .I........  
019W_W-M        .......... ....T..... .......... .......... .......... .........N .......... .I........  
0378B_W-M       .......... .......... .......... .......... .......... .........N .A........ .I........  
012OM_M         .......... .......... .......... .......... .......... .........N .A........ .I........  
0221AGD_K-P     .......... .......... .......... .......... .......... .........N .A........ .I........  
0222GD_K_P      .......... .......... .......... .......... .......... .........N .A........ .I........  
009B_W-M        .......... .......... .......... .......... .......... .........N .A........ .I........  
4W_W-M          .......... .......... .......... .......... .........L .........N .......... .I........  
015W_W-M        .......... .......... .......... .......... .........L .........N .......... .I........  
0741M_S         .......... .L........ .......... .......... .........L .........N .......... .I........  
0742M_S         .......... .L........ .......... .......... .........L .........N .......... .I........  
0131Z_S         .......... .L........ .......... .......... .........L .........N .......... .I........  
0133Z_S         .......... .L........ .......... .......... .........L .........N .......... .I........  
026Z_S          .......... .L........ .......... .......... .........L .........N .......... .I........  
020B_S          .......... .L........ .......... .......... .........L .........N .......... .I........  
042AK_K-P       .......... .L........ .......... .......... ...P.....L .........N .......... .I........  
0257G_W-M       .......... .......... .......... .......... .........L .........N .......... .I........  
0253G_W-M       .......... .......... .......... .......... .........L .........N .......... .I........  
0258G_W-M       .......... .......... .......... .......... .........L .........N .......... .I........  
014W_M          .......... ......H... .......... .......... .......... .........N .......... .I........  
017B_W-M        .......... .......... .......... .......... .......... .......... .......... ..........  
002B_W-M        .......... .......... .......... .......... .......... .......... .......... ..........  
001B_W-M        .......... .......... .......... .......... .......... .......... .......... ..........  
035S_P          .......... .......... .......... .......... .......... .......... .......... ..........  
0072B_W-M       .......... .......... .......... .......... .......... .......... .......... ..........  
00913B_W-M      .......... .......... .......... .......... .......... .......... .......... ..........  
0094B_W-M       .......... .......... .......... .......... .......... .......... .......... ..........  
0018P_Lodz      .......... .......... .......... .......... .......... .......... .......... ..........  
011L_Lodz       .......... .......... .......... .......... .......... .......... .......... ..........  
00111P_Lodz     .......... .......... .......... .......... .......... .......... .......... ..........  
01610BP_P       .......... ..F....... .......... .......... .......... .......... V......... ..........  
0169BP_P        .......... .......... .......... .......... .......... .......... V......... ..........  
0168BP_P        .......... .......... .......... .......... .......... .......... V......... ..........  
031W_W-M        .......... .......... .......... .......... .......... .......... .......... ..........  
BLV_FLK         .......... .......... .........R .......... .......... .........N .......R.. .I........  


                ----------------------------------CD8+ CTL epitopes-----
                Leucine-rich activation domain
                --------------------------------------                                                -
BLV_strain_344  LLPPFDTLLV DPLRLSVFAP DTRGAIRYLS TLLTLCPATC ILPLGEPFSP NVPICRFPRD TNEPPLSEFE LPLIQTPGLS 240 
0137O_L_S       .......... .......... .....T.... .......... .......... .......... S....P.... ..I.......  
011TL_L         .......... .......... .....T.... .......... .......... .......... S....P.... ..I.......  
0138O_L_S       .......... .......... .....T.... .......... .......... .......... S....P.... ..I.......  
0135O_L_S       .......... .......... .....T.... .......... .......... .......... S....P.... ..I.......  
0403W_W-M       .......... .......... .....T.... .......... .......... .......... S....P.... ..I.......  
10Sz_W-M        .......... ..P....... .......... .......... .......... .......... SD........ ..P.......  
11W_W-M         .......... ..P....... .......... .......... .......... .......... SD........ ..P.......  
0357M_P         .......... ..P....... .......... .......... .......... .......... SD........ ..P.......  
019L_P          .......... ..P....... .......... .......... .......... .......... SD........ ..P.......  
0355M_P         .......... ..P....... .......... .......... .......... .......... SD........ ..P.......  
019W_W-M        .......... ..P....... .......... .......... .......... .......... SD........ ..P.......  
0378B_W-M       .......... .......... .......... .......... .......... .......... .......... ..P.......  
012OM_M         .......... .......... .......... .......... .......... ...M...... .......... ..P.......  
0221AGD_K-P     .......... .......... .......... .......... .......... .......... .......... ..P.......  
0222GD_K_P      .......... .......... .......... .......... .......... .......... .......... ..P.......  
009B_W-M        .......... .......... .......... .......... .......... .......... .......... ..P.......  
4W_W-M          .......... .......... ..K....... .......... .......... .......... .......... ..........  
015W_W-M        .......... .......... ..K....... .......... .......... .......... .......... ..........  
0741M_S         .......... .......... ..K....... .......... .......... .......... .......... ..........  
0742M_S         .......... .......... ..K....... .......... .......... .......... .......... ..........  
0131Z_S         .......... .......... ..K....... .......... .......... .......... .......... ..........  
0133Z_S         .......... .......... ..K....... .......... .......... .......... .......... ..........  
026Z_S          .......... .......... ..K....... .......... .......... .......... .......... ..........  
020B_S          .......... .......... ..K....... .......... .......... .......... .......... ..........  
042AK_K-P       .......... .......... ..K....... .......... .......... .......... .......... ..........  
0257G_W-M       .......... .......... ..K....... .......... .......... .......... .......... ..........  
0253G_W-M       S......... .......... ..K....... .......... .......... .......... .......... ..........  
0258G_W-M       .......... .......... ..K....... .......... .......... .......... .......... ..........  
014W_M          .......... .......... .......... .......... .......... .......... .......... ..........  
017B_W-M        .......... .......... .......... .......... .......... .......... .......... ..........  
002B_W-M        .......... .......... .......... .......... .......... .......... .......... ..........  
001B_W-M        .......... .......... .......... .......... .......... .......... .......... ..........  
035S_P          .......... .......... .......... .......... .......... .......... .......... ..........  
0072B_W-M       .......... .......... .......... .......... .......... .......... .......... ..........  
00913B_W-M      .......... .......... .......... .......... .......... .......... .......... ..........  
0094B_W-M       .......... .......... .......... .......... .......... .......... .......... ..........  
0018P_Lodz      .......... .......... .......... .......... .......... .......... .......... ..........  
011L_Lodz       .......... .......... .......... .......... .......... .......... .......... ..........  
00111P_Lodz     .......... .......... .......... .......... .......... .......... .......... ..........  
01610BP_P       .......... .......... .......... .......... .......... .......... .......... ..........  
0169BP_P        .......... .......... .......... .......... .......... .......... .......... ..........  
0168BP_P        .......... .......... .......... .......... .......... .......... .......... ..........  
031W_W-M        .......... .......... .......... .......... .......... .......... .......... ..F.......  
BLV_FLK         ...S...... .......... .......... .......... .......... .......... S......... ..........  


                                      B-cell epitope
                                      ---------------------
                Multifunctional domain 
                ---------------------------                              +
BLV_strain_344  WSVPAIDLFL TGPPSPCDRL HVWSSPQALQ RFLHDPTLTW SELVASGKLR LDSPLKLQLL ENEWLSRLF 310
0137O_L_S       .......... .......... .......... .......... .......... .......... ......... 
011TL_L         .......... .......... ....G..... .......... .......... .......... ......... 
0138O_L_S       .......... .......... .......... .......... .......... .......... ......... 
0135O_L_S       .......... .......... .......... .......... .......... .......... ......... 
0403W_W-M       .......... .......... .......... .......... .......... .......... ......... 
10Sz_W-M        .......... ......G... .......... .......... P......... .......... ......... 
11W_W-M         .......... ......G... .......... .......... P......... .......... ......... 
0357M_P         .......... .......N.. .......... .......... P......... .......... ......... 
019L_P          .......... .......N.. .......... .......... P......... .......... ......... 
0355M_P         .......... .......N.. .......... .......... P......... .......... ......... 
019W_W-M        .......... .......... .......... .......... P......... .......... ......... 
0378B_W-M       .......... ......Y... .......... .......I.. .......... .......... ......... 
012OM_M         .......... ......Y... .......... .......... .......... .......... ......... 
0221AGD_K-P     .......... ......Y... .......... .......... .......... .......... ......... 
0222GD_K_P      .......... ......Y... .......... .......... .......... .......... ......... 
009B_W-M        .......... ......Y... .......... .......... .......... .......... ......... 
4W_W-M          .......... ......Y... .......... .......... .......... .......... ......... 
015W_W-M        .......... ......Y... .......... .......... .......... .......... ......... 
0741M_S         .......... ......Y... .......... .......... .......... .......... ......... 
0742M_S         .......... ......Y... .......... .......... .......... .......... ......... 
0131Z_S         .......... ......Y... .......... .......... .......... .......... ......... 
0133Z_S         .......... ......Y... .......... .......... .......... .......... ......... 
026Z_S          .......... ......Y... .......... .......... .......... .......... ......... 
020B_S          .......... ......Y... .......... .......... .......... .......... ......... 
042AK_K-P       .......... ......Y... .......... ...L...... .......... .......... ......... 
0257G_W-M       .......... ......Y... .......... .......... .......... .......... ......... 
0253G_W-M       .......... ......Y... .......... .......... .......... .......... ......... 
0258G_W-M       .......... ......Y... .......... .......... .......... .......... ......... 
014W_M          .......... ......Y... .......... .......... .......... .......... ......... 
017B_W-M        .......... .......... .......... .......... .......... .......... ......... 
002B_W-M        .......... ......F... .......... .......... .......... .......... ......... 
001B_W-M        .......... ......F... .......... .......... .......... .......... ......... 
035S_P          .......... .......... .......... .......... .......... .......... ......... 
0072B_W-M       .......... .......... .......... .......... .......... .......... ......... 
00913B_W-M      .......... .......... .......... .......... .......... .......... ......... 
0094B_W-M       .......... .......... .......... .......... .......... .......... ......... 
0018P_Lodz      .......... .......... .......... .......... .......... .......... ......... 
011L_Lodz       .......... .......... .......... .......... .......... .......... ......... 
00111P_Lodz     .......... .......... .......... .......... .......... .......... ......... 
01610BP_P       .......... .......... .......... .......... P......... .......... ......... 
0169BP_P        .......... .......... .......... .......... P......... .......... ......... 
0168BP_P        .......... .......... .......... .......... P......... .......... ......... 
031W_W-M        .......... .......... .......... .......... .......... .......... ......... 
BLV_FLK         .......... .......... .......... .......... ......R.I. .......... ......... 

Figure S3: Alignment of the translated amino acid sequences of Tax protein from forty-three Polish BLV strains. The points at which translated proviral Tax sequences differ from the reference are indicated below the strain 344 sequence. Functionally important regions include the following: Zn finger domain, phosphorylation sites, T-cell epitopes, B-cell epitope, Leucine-rich activation domain, Multifunctional domain, and CD8+ CTL epitopes which are identified with dashes lines. GenBank accession numbers for 43 new tax gene sequences are incrementally MT723750 to MT723792.


Figure S4: Phylogenetic analysis of Tax amino acid sequences. Phylogenetic relationship of a 310-aa fragment of 43 tax gene amino acid sequences, BLV-FLK, and strain 344 (pBLV344) (n = 45), as inferred by Bayesian analysis. Numbers at nodes indicate posterior probabilities of sampling the node among 10,000 trees. Tax variants selected for further analysis are indicated at the right by red squares
