# Supplementary material for: Effects of Naturally Occurring Mutations in Bovine Leukemia Virus 5′-LTR and Tax Gene on Viral Transcriptional Activity
Source: Pathogens. 2020 Oct 13;9(10):836. doi: 10.3390/pathogens9100836 (PMC7656303; doi:10.3390/pathogens9100836)
Supplement: Supplementary file 1 [file pathogens-09-00836-s001.zip › Tables.docx]

| Variant | pLTR Variant  without Tax  (*p*-value) | pLTR Variant  with pSGTax344  (*p*- value) | pLTR-WT  with pTax Variant  (*p*- value) | pLTR Variant  with Corresponding pTax Variant  (*p*- value) |
| --- | --- | --- | --- | --- |
| 011L | 0.0601 | **0.0051** | **0.0051** | **0.0006** |
| 00111P | **0.0093** | **0.0083** | **0.0251** | **0.0173** |
| 0094B | 0.8336 | 0.5353 | **0.0308** | **0.0164** |
| 0168BP | **0.0271** | **0.0083** | **0.0135** | **0.0004** |
| 01610BP | **0.0014** | **0.0214** | **0.0135** | **0.0021** |
| 031W | 0.1118 | 1.0000 | **0.0131** | **0.0016** |
| 001B | 0.0657 | **0.0032** | **0.0022** | 0.3077 |
| 0137O | 0.2713 | 0.6745 | 0.1738 | 0.1118 |
| 011TL | 0.2891 | 0.5755 | **0.0375** | 0.1499 |
| 11W | 0.5287 | 0.9203 | **0.0121** | **0.0085** |
| 012OM | 0.3125 | 0.5287 | 0.0658 | **0.0102** |
| 019L | **0.0019** | **0.0121** | **0.0121** | **0.0004** |
| 10Sz | 0.9203 | 0.0601 | **0.0214** | **0.0080** |
| 014W | 0.0548 | 0.1164 | **0.0455** | **0.0009** |
| 0741M | 0.0891 | **0.0324** | **0.0015** | **0.0001** |
| BLV-FLK | 0.1285 | 0.0969 | **0.0271** | 0.0836 |

**Table S1:** Differences in luciferase activity level between the 344 strain and different LTR and Tax variants.

Data were analyzed by nonparametric Mann-Whitney U test, *p*-values indicating significant differences between groups were reported in the table. A significant *p* values are bold.

**Table S2:** Identity and origin of the sequences analysed in the study.

| **GenBank Accession No** | **Geographic Origin & Voivodeship** | **Genotype** | **Identity Code & Source** |
| --- | --- | --- | --- |
| MT740097 | Poland: Warmian-Masurian | 4-II | 0258G_W-M, this work |
| MH407735 | Poland: Warmian-Masurian | 4-II | 0253G_W-M, Pluta, 2018 |
| MT740098 | Poland: Warmian-Masurian | 4-II | 0257G_W-M, this work |
| MH407736 | Poland: Warmian-Masurian | 4-II | 0252G_W-M, Pluta, 2018 |
| MT740099 | Poland: Kuyavian-Pomeranian | 4-II | 042AK_K-P, this work |
| MH423657 | Poland: Warmian-Masurian | 4-II | 0242K_W-M, Pluta, 2018 |
| MT740100 | Poland: Warmian-Masurian | 4-II | 0256G_W-M, this work |
| MT740101 | Poland: Warmian-Masurian | 4-II | 0251G_W-M, this work |
| MT740102 | Poland: Warmian-Masurian | 4-II | 0255G_W-M, this work |
| MH407742 | Poland: Silesian | 4-II | 0741M_S, Pluta, 2018 |
| MT740103 | Poland: Silesian | 4-II | 0133Z_S, this work |
| MH407740 | Poland: Silesian | 4-II | 0131Z_S, Pluta, 2018 |
| MT740104 | Poland: Silesian | 4-II | 0742M_S, this work |
| MH407741 | Poland: Silesian | 4-II | 026Z_S, Pluta, 2018 |
| MH407744 | Poland: Silesian | 4-II | 020B_S, Pluta, 2018 |
| MT740105 | Poland: Silesian | 4-II | 0132Z_S, this work |
| MH407743 | Poland: Silesian | 4-II | 053K_S, Pluta, 2018 |
| MH423641 | Poland: Warmian-Masurian | 4-IIIc | 0371B_W-M, Pluta, 2018 |
| MH423642 | Poland: Warmian-Masurian | 4-IIIc | 0374B_W-M, Pluta, 2018 |
| MH423643 | Poland: Warmian-Masurian | 4-IIIc | 0378B_W-M, Pluta, 2018 |
| MH423669 | Poland: Warmian-Masurian | 4-IIIa | 3208M_W-M, Pluta, 2018 |
| MT740106 | Poland: Warmian-Masurian | 4-IIIa | 3175aM_W-M, this work |
| MT740107 | Poland: Warmian-Masurian | 4-IIIa | 3205M_W-M, this work |
| MT740108 | Poland: Warmian-Masurian | 4-IIIa | 3176aM_W-M, this work |
| MT740109 | Poland: Masurian | 4-IIIa | 012OM_M, this work |
| MH423659 | Poland: Lodz | 4-IIIa | 047P_Lodz, Pluta, 2018 |
| MH423661 | Poland: Kuyavian-Pomeranian | 4-IIIb | 0222GD_K-P, Pluta, 2018 |
| MH423660 | Poland: Kuyavian-Pomeranian | 4-IIIb | 0221AGD_K-P, Pluta, 2018 |
| MT740110 | Poland: Kuyavian-Pomeranian | 4-IIIb | 0221GD_K-P, this work |
| MT740111 | Poland: Masurian | 4-II | 014W_M, this work |
| MH423646 | Poland: Kuyavian-Pomeranian | 4-Ia | 014NN_K-P, Pluta, 2018 |
| MH423636 | Poland: Podlaskie | 4-Ia | 0166BP_P, Pluta, 2018 |
| MH423637 | Poland: Podlaskie | 4-Ia | 01610BP_P, Pluta, 2018 |
| MH423634 | Poland: Podlaskie | 4-Ia | 0168BP_P, Pluta, 2018 |
| MT740112 | Poland: Podlaskie | 4-Ia | 0169BP_P, this work |
| MT740113 | Poland: Masurian | 4-Ia | 022WM_M, this work |
| MH423635 | Poland: Podlaskie | 4-Ia | 0167BP_P, Pluta, 2018 |
| MH423654 | Poland: Warmian-Masurian | 4-Ia | 4W_W-M, Pluta, 2018 |
| MH423655 | Poland: Warmian-Masurian | 4-Ia | 0071B_W-M, Pluta, 2018 |
| MT740114 | Poland: Warmian-Masurian | 4-Ia | 015W_W-M, this work |
| MH423644 | Poland: Warmian-Masurian | 4-Ia | 009B_W-M, Pluta, 2018 |
| MT740115 | Poland: Warmian-Masurian | 4-Ia | 0362B_W-M, this work |
| MH407738 | Poland: Podlaskie | 4-Ia | 035S_P, Pluta, 2018 |
| MH423645 | Poland: Warmian-Masurian | 4-Ia | 006B_W-M, Pluta, 2018 |
| MT740116 | Poland: Warmian-Masurian | 4-I | 0244K_W-M, this work |
| MH423633 | Poland: Greater Poland | 4-I | 015P_G_P, Pluta, 2018 |
| MT740117 | Poland: Warmian-Masurian | 4-I | 031W_W-M, this work |
| MH423650 | Poland: Warmian-Masurian | 4-I | 017B_W-M, Pluta, 2018 |
| MH423640 | Poland: Warmian-Masurian | 4-I | 038W_W-M, Pluta, 2018 |
| MT740118 | Poland: Lodz | 4-Id | 00111P_Lodz, this work |
| MT740119 | Poland: Lodz | 4-Id | 0018P_Lodz, this work |
| MT740120 | Poland: Lodz | 4-Id | 0057P_Lodz, this work |
| MT740121 | Poland: Lodz | 4-Id | 011L_Lodz, this work |
| MT740122 | Poland: Warmian-Masurian | 4-Id | 0378B17_W-M, this work |
| MT740123 | Poland: Warmian-Masurian | 4-Ib | 001B_W-M, this work |
| MT740124 | Poland: Warmian-Masurian | 4-Ib | 002B_W-M, this work |
| MH423656 | Poland: Warmian-Masurian | 4-Ic | 0072B_W-M, Pluta, 2018 |
| MH423649 | Poland: Warmian-Masurian | 4-Ic | 0102B_W-M, Pluta, 2018 |
| MH423668 | Poland: Warmian-Masurian | 4-I | 030O_W-M, Pluta, 2018 |
| MT740125 | Poland: Warmian-Masurian | 4-Ie | 0097B_W-M, this work |
| MT740126 | Poland: Warmian-Masurian | 4-Ie | 0099B_W-M, this work |
| MT740127 | Poland: Warmian-Masurian | 4-Ie | 00912B_W-M, this work |
| MT740128 | Poland: Warmian-Masurian | 4-Ie | 0092B_W-M, this work |
| MT740129 | Poland: Warmian-Masurian | 4-Ie | 00911B_W-M, this work |
| MT740130 | Poland: Warmian-Masurian | 4-Ie | 00914B_W-M, this work |
| MH423648 | Poland: Warmian-Masurian | 4-Ie | 0101B_W-M, Pluta, 2018 |
| MT740131 | Poland: Warmian-Masurian | 4-Ie | 0095B_W-M, this work |
| MT740132 | Poland: Warmian-Masurian | 4-Ie | 00910B_W-M, this work |
| MH423647 | Poland: Warmian-Masurian | 4-Ie | 0094B_W-M, Pluta, 2018 |
| MT740133 | Poland: Warmian-Masurian | 4-Ie | 0093B_W-M, this work |
| MT740134 | Poland: Warmian-Masurian | 4-Ie | 0096B_W-M, this work |
| MT740135 | Poland: Warmian-Masurian | 4-Ie | 0098B_W-M, this work |
| MT740136 | Poland: Warmian-Masurian | 4-Ie | 00913B_W-M, this work |
| MH423652 | Poland: Lublin | 8-I | 011TL_L, Pluta, 2018 |
| MT740137 | Poland: Podlaskie | 8-I | 0083Z_P, this work |
| MH423666 | Poland: Podlaskie | 8-I | 019WM_P, Pluta, 2018 |
| MH423651 | Poland: Podlaskie | 8-I | 0081Z_P, Pluta, 2018 |
| MH423639 | Poland: Warmian-Masurian | 8-I | 010W_W-M, Pluta, 2018 |
| MT740138 | Poland: Warmian-Masurian | 8-I | 0409W_W-M, this work |
| MT740139 | Poland: Warmian-Masurian | 8-I | 04010W_W-M, this work |
| MT740140 | Poland: Warmian-Masurian | 8-I | 0402W_W-M, this work |
| MH423653 | Poland: Warmian-Masurian | 8-I | 0405W_W-M, Pluta, 2018 |
| MT740141 | Poland: Warmian-Masurian | 8-I | 0403W_W-M, this work |
| MT740142 | Poland: Warmian-Masurian | 8-I | 0408W_W-M, this work |
| MT740143 | Poland: Warmian-Masurian | 8-I | 0407W_W-M, this work |
| MH423662 | Poland: Lower Silesian | 8-I | 0139O_L_S, Pluta, 2018 |
| MT740144 | Poland: Lower Silesian | 8-I | 0136O_L_S, this work |
| MT740145 | Poland: Lower Silesian | 8-I | 0138O_L_S, this work |
| MT740146 | Poland: Lower Silesian | 8-I | 01310O_L_S, this work |
| MT740147 | Poland: Lower Silesian | 8-I | 0137O_L_S, this work |
| MH423664 | Poland: Lower Silesian | 8-I | 0133O_L_S, Pluta, 2018 |
| MT740148 | Poland: Lower Silesian | 8-I | 0134O_L_S, this work |
| MH423665 | Poland: Lower Silesian | 8-I | 0132O_L_S, Pluta, 2018 |
| MH423663 | Poland: Lower Silesian | 8-I | 0135O_L_S, Pluta, 2018 |
| MT740149 | Poland: Warmian-Masurian | 7-I | 11W_W-M, this work |
| MH423667 | Poland: Podlaskie | 7-I | 0184S_P, Pluta, 2018 |
| MT740150 | Poland: Podlaskie | 7-I | 03510M_P, this work |
| MT740151 | Poland: Podlaskie | 7-I | 0355M_P, this work |
| MH423658 | Poland: Podlaskie | 7-I | 03513M_P, Pluta, 2018 |
| MT740152 | Poland: Podlaskie | 7-I | 0183S_P, this work |
| MT740153 | Poland: Podlaskie | 7-I | 0356M_P, this work |
| MH748226 | Poland: Podlaskie | 7-I | 03511M_P, Pluta, 2018 |
| MT740154 | Poland: Podlaskie | 7-I | 0357M_P, this work |
| MT740155 | Poland: Warmian-Masurian | 7-I | 10Sz_W-M, this work |
| MH423638 | Poland: Warmian-Masurian | 7-I | 019W_W-M, Pluta, 2018 |
| MT740156 | Poland: Podlaskie | 7-I | 019L_P, this work |
| EF600696.1 | USA, subclone pBLV913 | I | BLV_FLK, Derse, 1985 |
| JC613347.1 | Belgium | 4-Ia | pBLV344 |

**Table S3.** Sequences of primers used to amplify, sequence and clone the BLV LTR and tax regions.

| Fragment of BLV Genome | Name | Location | Sequence |
| --- | --- | --- | --- |
| LTR^ײַ^ | Primers used for overlap extension PCR^§^ | | |
|  | Amplification 979 bp fragment | | |
|  | P7736 | 7736–7756 | 5′- TCGATACCCTCCTTGTGGACC-3′ |
|  | P8693 | 8693–8714 | 5′-TGTTTGCCGGTCTCTCCTGGCC-3′ |
|  | Amplification 571 bp fragment | | |
|  | P1 | 1–20 | 5′-TGTATGAAAGATCATGCCGA-3′ |
|  | P609 | 591–609 | 5′- GACCCAAAATGCCGCCGAG-3′ |
|  | Primers used for cloning | | |
|  | P8169 | 8169–8192 | 5′-TATAGCTAGCTTGAGGGGGAGTCATTTGTATGAA-3′^*^ |
|  | P520 | 520–541 | 5′-TATAAGCTTGCGCCCCCAATTGTTTGCCGGT-3′^*^ |
|  | Primers used for colony PCR^¥^ | | |
| pMiniT | Cloning Analysis Forward Primer | 398–418 | 5′-ACCTGCCAACCAAAGCGAGAAC-3′ |
|  | Cloning Analysis Reverse Primer | 683–706 | 5′-TCAGGGTTATTGTCTCATGAGCG-3′ |
|  | Primers used for sequencing |  |  |
| pGL4.11 | Reporter Vector primer 3 (RVprimer3) ^Գ^ | 4319–4338 | 5′-TAGCAAAATAGGCTGTCCCC-3′ |
| LTR^ײַ^ | P509 ^Գ^ | 509-530 | 5′-TGTTTGCCGGTCTCTCCTGGCC-3′ |
| Tax^ײַ^ | Primers used for nested PCR | | |
|  | Amplification 2746 bp fragment | | |
|  | P5565 | 5565–5583 | 5′-TGGGTCAACTCGTCCTCGT-3′ |
|  | P8292 | 8292–8310 | 5′-AGGGGAAGTTGGGGAGGTA-3′ |
|  | Amplification 1019 bp fragment | | |
|  | P7206 ^Գ^ | 7206–7221 | 5′-CCATTACCTGATAACG-3′ |
|  | P8212 ^Գ^ | 8212–8224 | 5′-GGGCGGTGGCGGC-3′ |
|  | Primers used for cloning | | |
|  | P7199 | 7199–7224 | 5′- TCGGGATCCATTACCTGATAACGACA-3′ |
|  | P8213 | 8213–8224 | 5′-TGAGCTCGGGCGGTGGCGG-3′^*^ |
|  | Primers used for colony PCR^¥^ | | |
| pSG5(modified) | T7 | 1022–1040 | 5′- TAATACGACTCACTATAGG-3′ |
| Tax^ײַ^ | P8213 | 8213–8224 | 5′-TGAGCTCGGGCGGTGGCGG-3′ |
|  | Primers used for sequencing | | |
| Tax^ײַ^ | P7806 ^Գ^ | 7806-7825 | 5′- GCGTCAAAAGGGTGGAGAGA-3′ |
| pSG5(modified) | P1101 ^Գ^ | 1101-1130 | 5′-GTGAAATTTGTGATGCTATTGCTTTATTTG-3′ |

^§^ The technique of the overlap extension PCR was described by Pluta et al., 2018. ^ײַ^Numbering of genomic locations is based on the first published complete BLV genome (Sagata et al., 1985; GenBank accession no. K02120).^Գ^ Primers used for sequencing. ^*^ Primers, in which the underlined sequences correspond to restriction sites for NheI, HindIII, SacI.^¥^ Screening for inserts was performed by colony PCR according to the manufacturer's recommendations, by use of the OptiTaq DNA Polymerase (EURx).
